# Downregulation of SFRP2 facilitates cancer stemness and radioresistance of glioma cells via activating Wnt/β-catenin signaling

**DOI:** 10.1371/journal.pone.0260864

**Published:** 2021-12-01

**Authors:** Quansheng Wu, Xiaofeng Yin, Wenbo Zhao, Wenli Xu, Laizhao Chen

**Affiliations:** Department of Neurosurgery, Second Hospital of Shanxi Medical University, Taiyuan, Shanxi Province, China; Università degli Studi della Campania, ITALY

## Abstract

Secreted frizzled-related protein 2 (SFRP2) is a glycoprotein with frizzled-like cysteine-rich domain that binds with Wnt ligands or frizzled receptors to regulate Wnt signaling. SFRP2 is frequently hypermethylated in glioma patients, and analysis of TCGA data indicates that SFRP2 is one of the most downregulated genes in radiotherapy treated glioma patients. In the present study, we aimed to explore the potential function of SFRP2 in tumorigenesis and radioresistance of glioma. The RNA sequencing data of TCGA glioma samples were downloaded and analyzed. SFRP2 expression in 166 glioma patients was evaluated by qRT-PCR. The potential functions of SFRP2 in glioma were evaluated by loss-of-function assays and gain-of-function assays in glioma cell lines. We found that SFRP2 was downregulated in radiotherapy-treated glioma patients, and low SFRP2 expression was correlated with advanced tumor stage and poor prognosis. CRISP/Cas9-meidated SFRP2 knockdown promoted soft agar colony formation, cancer stemness and radioresistance of glioma cells, while enforced SFRP2 expression exhibited opposite effects. Moreover, Wnt/β-catenin signaling was activated in radiotherapy treated glioma patients. SFRP2 knockdown activated Wnt/β-catenin signaling in glioma cell lines, while overexpression of SFRP2 inhibited Wnt/β-catenin activation. Besides, pharmacological inhibition of Wnt/β-catenin signaling by XAV-939 abrogated the effects of SFRP2 knockdown on cancer stemness and radioresistance of glioma cells. Our data for the first time demonstrated a role of SFRP2 in radioresistance of glioma cells, and suggested that inhibition of Wnt/β-catenin signaling might be a potential strategy for increasing radiosensitivity of glioma patients.

## Introduction

Glioma arises from glia, the supporting tissue of brain. It is the most common and aggressive form of brain cancer, accounting for more than 80% malignant tumors in the central nervous system [1]. According to the World Health Organization (WHO) classification, glioma can be classified into different histologic types such as astrocytoma (grade I-IV), oligodendroglioma (grade II-III) and oligoastrocytoma (grade II-III) [2]. Glioblastoma multiforme (GBM, grade IV astrocytoma) is the most common (>50%) and malignant form of glioma [2]. The age-adjusted incidence for all gliomas is 4.67 to 5.73 per 100 000 persons [3]. Survival of glioma patients varies greatly by histologic type and grade. For instance, the survival for low-grade gliomas (grade I-II) is 5 to 15 years, and 2 to 3 years for grade III gliomas [4]. Glioblastoma has the poorest prognosis among all gliomas, with a median survival of 15 months and 5-year survival rate less than 5% [5]. The treatment options for high-grade gliomas include maximal surgical resection, radiotherapy and chemotherapy. Radiotherapy is the first treatment that proves to be effective in high-grade gliomas by randomized trials [6]. However, the effect of radiotherapy is limited due to intrinsic or acquired resistance of glioma cells. Thus, a thoroughly understanding of the molecular mechanism of glioma radioresistance might help to develop novel strategies to improve radiosensitivity.

Accumulated studies indicate that existing of glioma stem cells and activation of Wnt/β-catenin signaling might contribute to radioresistance of glioma cells [7]. Cancer stem cells are a subset of tumor cells that possess the ability to self-renew and differentiate into other types of tumor cells [8]. The existence of glioma stem cells (GSCs) are proved by a variety of studies [9,10]. In addition, GSCs are demonstrated resisting to radiotherapy [11]. For example, overexpression of PAF promotes GSCs maintenance and self-renewal, thus enhances radioresistance of glioma cells [12]. CD133 is a candidate marker for GSCs. There is evidence that CD133+ glioma cells have survival advantages than CD133- glioma cells after radiation [13]. Wnt/β-catenin signaling plays an important role in regulating self-renewal and differentiation during the development of central nervous system. Aberrant activation of Wnt/β-catenin signaling is involved in the tumorigenesis and chemo-radioresistance of glioma [14]. Wnt/β-catenin signaling is also vital for the maintenance of GSCs [14]. In addition, activation of Wnt/β-catenin signaling is reported to promote radioresistance of glioma cells, too. For example, cyclophilin A facilitates stemness, self-renewal and radioresistance of GSCs by activating Wnt/β-catenin signaling [15].

The secreted frizzled-related protein (SFRP) family has five members: SFRP1, SFRP2, SFRP3, SFRP4 and SFRP5. These members are glycoproteins with a frizzled-like cysteine-rich domain, thus can bind with Wnt ligands or frizzled receptors to regulate Wnt signaling [16]. Secreted frizzled-related protein 2 (SFRP2), a key member of the SFRP family, can act as antagonist or agonist for Wnt signaling, which seems to be context dependent [17]. In mesenchymal stem cells, SFRP2 overexpression inhibits Wnt signaling by decreasing β-catenin level [18]. In contrast, SFRP2 is proved to activate β-catenin signaling through Frizzled receptors in mouse intestinal epithelium [19]. SFRP2 can act as tumor suppressor in cancers, including glioma. SFRP2 is downregulated in glioma patients by promoter hypermethylation [20]. In addition, low SFRP2 expression predicts poor survival of glioblastoma patients, while SFRP2 overexpression reduced tumor growth in mice [21]. However, the potential function of SFRP2 in radioresistance of glioma patients has not been studied before. In the present study, we found that SFRP2 was downregulated in radiotherapy treated glioma patients, and low SFPR2 expression was correlated with advanced tumor stage and poor prognosis. SFRP2 knockdown promoted soft agar colony formation, cancer stemness and radioresistance of glioma cells, while overexpression of SFRP2 exhibited contrary effects. Moreover, SFRP2 regulated Wnt/β-catenin activation in glioma cells, and pharmacological inhibition of Wnt/β-catenin signaling by XAV-939 abolished the effects of SFRP2 knockdown in glioma cells. Our results for the first time demonstrated that SFRP2 was involved in radioresistance of glioma, and inhibition of Wnt/β-catenin signaling might increase the effect of radiotherapy in glioma patients.

## Materials and methods

### Patient samples

This study was reviewed and approved by the Ethics Committee of the Second Hospital of Shanxi Medical University. All procedures involved in human participants were performed according to the guidelines with the Declaration of Helsinki and Ethics Committee of the Second Hospital of Shanxi Medical University. All human participants knew the study concept, agreed to participant, and signed the written informed consents. A total of 166 pairs of glioma specimens and adjacent normal brain tissues were collected from the Second Hospital of Shanxi Medical University between May 2014 and March 2016. The pathologic diagnosis of glioma was conducted by two pathologists independently. Tumor grade of glioma patients was staged according to the WHO classification. In our study, 45 glioma patients were WHO grade II, 60 WHO grade III and 61 WHO grade IV. Besides, 88 of all 166 glioma patients had previously treated with radiotherapy. The tissue samples were snap frozen and stored in liquid nitrogen until use. The demographic and clinicopathological features of glioma patients were obtained from the hospital records. All glioma patients in our study were followed up for as long as 48 months after surgery.

### Cell culture and reagents

Glioma cell lines SW1783, U87MG, SW1088, LN18, T98G and U251MG, and human HEK293T cells were purchased from the American Type Culture Collection (ATCC). Glioma cell lines GMS-10 and GOS-3 were obtained from Deutsche Sammlung von Mikroorganismen und Zellkulturen (DSMZ). All cell lines were cultured in high glucose Dulbecco’s modified Eagle’s medium (DMEM, Thermo Fisher Scientific, USA) medium supplemented with 10% fetal bovine serum (FBS, Hyclone, USA), 100 units/mL penicillin and 100 units/mL streptomycin in a humidified atmosphere at 37 ˚C with 5% CO_2_. XAV-939 (#S1180) was purchased from Selleck Chemicals (USA) and dissolved in dimethylsulfoxide (DMSO).

### Plasmid constructs and lentivrius infection

The coding sequences of SFRP2 were inserted into the pCDH lentivirus vector (System Biosciences #CD510B-1) to build the SFRP2 expression lentivirus. The empty pCDH lentivirus vector was regarded as empty vector (EV) control. Two short guide RNAs (sgRNAs) targeting SFRP2 (sg-SFRP2-1 and sg-SFRP2-2) were designed and inserted into the lentiCRISPRv2 vector (Addgene #52961, USA). A non-targeting sequence was inserted into the lentiCRISPRv2 vector to construct the sg-NC control. Lentivirus particles were produced by co-transfecting HEK293T cells with lentivirus plasmid and helper plasmids pCMV-VSV-G, pRSV-REV and pMDL using lipofectamine 3000 (Invitrogen, USA). Lentivirus particles were collected at 24, 48 and 72 h after transfection and filtered through 0.22 μm filter. Lentivirus infection was conducted by incubating 1 mL virus-containing medium (about 5 × 10^7^ lentivirus particles) with 1 × 10^6^ cells supplemented with 8 μg/mL polybrene (Sigma, USA) overnight. Transient transfection was performed using lipofectamine 3000 (Invitrogen, USA) according to the manufacturer’s instructions. The sequences for SFRP2 sgRNAs were: sg-SFRP2-1, 5’-TGAGT GCGAC CGTTT CCCCC-3’; sg-SFRP2-2, 5’-GCCCC GGTCA TGTCC GCCTT-3’; sg-NC, 5’-ACGGA GGCTA AGCGT CGCAA-3’.

### TCGA data analysis

The RNA sequencing data for the TCGA glioma tissues and paired normal samples were downloaded from the GDC Data Portal (https://gdac.broadinstitute.org/). PRADA tool was used to align the RNA-sequencing data. HtSeq V0.6.1 was used to evaluate the RNA sequencing reads. Limma package (version: 3.40.2) of R software was used to identify differentially expressed mRNAs between radiotherapy treated and non-radiotherapy treated glioma patients. The differentially expressed mRNAs were defined as adjusted *p* < 0.05 and |log_2_ Fold Change| ≥ 1.

### Quantitative real-time polymerase chain reaction (qRT-PCR)

Total RNAs from tissue samples and culture cells were extracted using the Qiagen RNA extraction kit (Qiagen, Germany) as protocol indicated. Transcriptor First Strand cDNA Synthesis Kit (Roche, Germany) was used to synthesize the complementary DNA from RNA samples. SYBR Premix ex Taq™ II kit (Takara, Japan) was used to perform qPCR analysis on a Roche LightCycler system (Roche, Germany). Relative gene expression was measured using the 2^-ΔΔCq^ method and normalized to GAPDH. The primer sequences were: SFRP2, sense: 5’-GCCAC GGCAT CGAAT ACCAG AA-3’, antisense: 5’-CGAAG AGCGA GCACA GGAAC TT-3’; Oct4, sense: 5’-GCAAG CGATC AAGCA GCGAC TA-3’, antisense: 5’-ACCGA GGAGT ACAGT GCAGT GA-3’; Lin28, sense: 5’-GATTC TCCTG CCTCA GCCTC CT-3’, antisense: 5’-CCAGC CTGGA CAACA TGGTG AA-3’; Nanog, sense: 5’-TGGAG GGTGG AGTAT GGTTG GA-3’, antisense: 5’-AGGCA GGAGA ATGGC GTGAA C-3’; Sox2, sense: 5’-GTACT GGCGA ACCAT CTCTG TG-3’, antisense: 5’-TACCA ACGGT GTCAA CCTGC AT-3’. All samples were done with three repeats.

### Immunohistochemistry (IHC)

Tissue sections (5 μm) were deparaffinized by xylene, and rehydrated by alcohol gradients. Citrate buffer (pH 6.0) was used for antigen retrieval by microwaving heating. Endogenous peroxidase was eliminated by 3% H_2_O_2_ in methanol for 30 min. The slides were blocked with 10% goat serum, and incubated with specific antibodies at 4 ˚C overnight. Next, the slides were stained with horseradish peroxidase anti-rabbit antibody for 1 h at room temperature. The DAB Plus Substrate Detection System (Thermo Fisher Scientific, USA) was used to for signal detection. Images were photographed by a Nikon ECLIPSE E800 fluorescence microscope. The specific antibodies were: Anti-SFRP2 rabbit IgG (Abcam#ab137560, 1: 300) and Active β-Catenin Rabbit mAb (Cell signaling#19807, 1: 300).

### X-ray irradiation

X-ray irradiation was conducted by MBR-1505R2 (Hitachi, Tokyo, Japan). The irradiation condition was 150 kV voltage, 5 mA current and 0.5 Gy/min at the target. For glioma cell irradiation, SW1783, SW1088, LN18 and T98G cells were treated with a single dose of 2 or 4 Gy X-ray irradiation at room temperature. For animal experiment, mice were anesthetized by barbiturate and immobilized in a customized harness. The mice body was shielded by lead to minimize the amount of normal tissue irradiated. Tumor xenografts received a 2 Gy dose of X-rays daily for three weeks.

### Cell viability assay

The viability of cells was measured by Cell Counting Kit-8 (Beyotime, China) as protocol indicated. Briefly, cells were seeded in 96-well plates (3000/well) for 24 h and received a single dose of 2 or 4 Gy X-ray irradiation. Viability of cells was evaluated at 72 h post-irradiation by incubating cells with 10 μL CCK8 reagent per well at 37 ˚C for 2 h. Then the plates were rotated on a microplate shaker for 3 min. The absorption at 450 nm was detected by a microplate reader. Each sample had three repeats at same time.

### Western blot

Protein lysates from tissue samples or culture cells were extracted by RIPA lysis buffer (Beyotime, China) with protease inhibitors (Sigma, USA). The protein concentration was determined by BCA kit (Sigma, USA). A total of 30 μg proteins were loaded to each well and separated by 10% or 15% SDS-PAGE. Next, proteins were transferred to nitrogen membranes (Thermo Fisher Scientific, USA) and blocked by 5% non-fat milk for 1 h at room temperature. The membranes were stained with specific first antibodies at 4 ˚C overnight and corresponding second antibodies for 1 h at room temperature. The antibodies were: Anti-SFRP2 rabbit IgG (Abcam#ab137560, 1: 1000), Cleaved Caspase-3 Rabbit mAb (Cell signaling#9664, 1: 1000), GAPDH Rabbit mAb (Cell signaling#5174 1: 1000), β-Catenin Rabbit mAb (Cell signaling#8480, 1: 1000), Active β-Catenin Rabbit mAb (Cell signaling#19807, 1: 1000), GSK-3β Rabbit mAb (Cell signaling#12456, 1: 1000), Phospho-GSK-3β (Ser9) Rabbit mAb (Cell signaling#5558, 1: 1000) and goat anti-rabbit IgG-HRP (Cell signaling #7074; 1:4000).

### Soft agar assay

Soft agar colony formation assay was performed according to previous report [22]. In brief, cells were dispersed as single cell suspension by digesting with 0.05% trypsin. Then, cells were seeded in 0.4% top agar at a density of 8000 cells/well in 6-well plates. The bottom agar was 0.6%. The cells were cultured for three weeks until visible colonies achieved. Next, the colonies were incubated with 0.5 mL thiazolyl blue tetrazolium bromide solution (MTT, 1 mg/mL, Sigma-Aldrich, USA) at 37 ˚C for 2 h. Images of colonies were obtained by a scanner. All samples were done with three repeats.

### Sphere formation assay

Cells were dispersed as single cell suspension by 0.05% trypsin, then seeded in 6-well ultra-low attachment plates (Corning, USA) at a density of 1000 cells/well. The culture medium was supplemented with 2% B27, 10 ng/ml EGF and 10 ng/ml FGF. Next, cells were subcultured at a density of 1000 cells/well once every week to develop secondary or tertiary spheres. All samples were done with three repeats.

### TUNEL staining

The apoptotic cells were detected by One step TUNEL apoptosis assay kit (Beyotime #C1090, China) according to manufacturer’s instructions. Briefly, cells were washed with PBS for twice, and fixed with 4% paraformaldehyde for 15 min at room temperature. 0.25% Triton-X 100 was used for permeabilizing cells for 30 min at room temperature. Next, cells were stained with terminal dexynucleotidyl transferase (TdT) reaction cocktail for 60 min at 37 ˚C in a dark room. The nucleus was stained with DAPI for 10 min at room temperature. Washed the cells with PBS for three times, and photographed the cells by a Leica SP8 confocal microscope. All samples were done with three repeats.

### Flow cytometry

Cells were digested as single cell suspensions by 0.05% trypsin. Next, 1 × 10^6^ cells were stained with Annexin V-FITC and propidium iodide (PI) for 15 min at room temperature in a dark room to evaluate apoptotic cells. Cells were stained with CD133 (Cells signaling #60577, 1: 100) to evaluate cancer stemness. Detected the fluorescence signal at 488/530 by a LSR Fortessa flow cytometer (BD Biosciences, USA) and analyzed the data by Flowjo 7.6 software. All samples were done with three repeats.

### Tumor xenograft growth model

The animal experiments were reviewed and approved by the Animal Care and Experimental Committee of the Second Hospital of Shanxi Medical University. SW1783 cells (2 × 10^6^) were infected with sg-SFRP2-1 or sg-NC lentiviral particles, then subcutaneously injected into six-week old BABL/c nude mice (about 20 g). BABL/c nude mice were housed in individually ventilated cages under specific pathogen free conditions. Mice were allowed access to sterilized water and feed *ad libitum*. Tumor xenografts were allowed to grow for 7 d, then mice were randomly divided into eight groups (n = 4 for each group) as depicted in Figs 2L and 6I. Mice were treated with 2 Gy X-ray irradiation, XAV-939 (25 mg/kg) or equal volume of Veh control (50% PEG-400) daily for three weeks as indicated. XAV-939 was dissolved in 50% PEG-400 (PEG-400: PBS = 1: 1). The length and width of the tumor was measured by caliper once every three days. Tumor volume was calculated according to the formula: Volume = (length×width^2^/2). At the end of treatment, mice were anaesthetized by 3% isoflurane and sacrificed by broking the neck. Tumor xenografts were dissected out, photographed, and weighed. In the animal experiment, all tumor volumes were less than 2000 mm^3^ and larger than 100 mm^3^. The tumor lengths in each dimension were less than 2 cm.

### Statistical analysis

Statistical analysis was performed by the SPSS 19.0 and GraphPad Prism 8.0 software. Two-tailed Student’s t test was used to compare difference between two groups. One-way ANOVA (Tukey’s post-hoc test) was used to compare difference among multiple groups. Clinical variables was evaluated by Pearson Chi-squared test. The data was exhibited as mean ± standard deviation (x ± s.d). *P*< 0.05 was regarded as statistically significant.

## Results

### SFRP2 is downregulated in radiotherapy treated glioma patients and predicts poor prognosis

**Fig 1:**
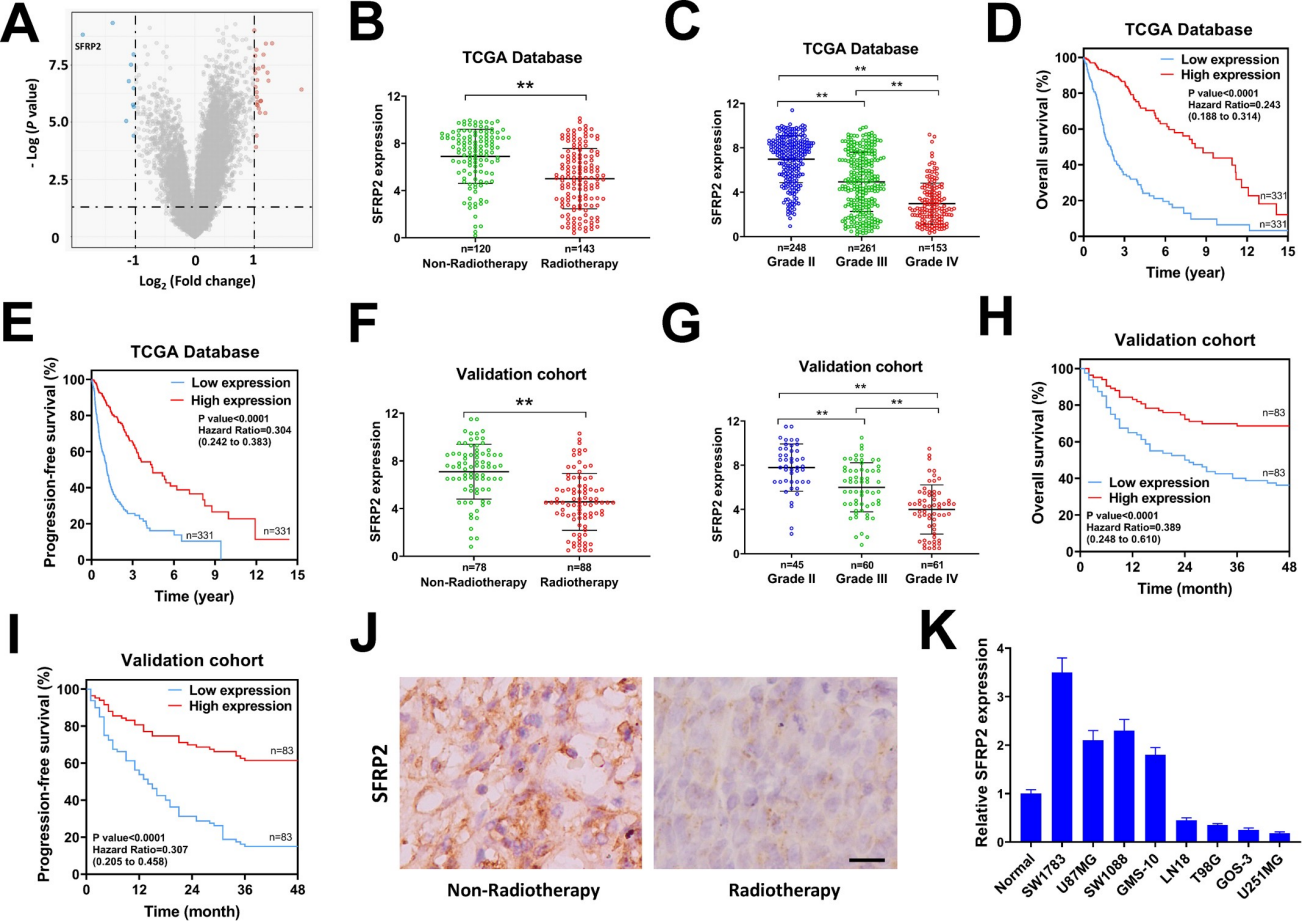
SFRP2 is downregulated in radiotherapy treated glioma patients and predicted poor prognosis. A, the differentially expressed mRNAs in radiotherapy-treated glioma patients versus non-radiotherapy treated glioma patients in TCGA were depicted in the volcano plots. B, SFRP2 expression in radiotherapy-treated glioma patients and non-radiotherapy treated glioma patients in TCGA. C, SFRP2 expression in WHO grade II, III and IV glioma patients in TCGA. D, overall survival of glioma patients according to SFRP2 expression in TCGA. E, progression-free survival of glioma patients according to SFRP2 expression in TCGA. F, SFRP2 expression in radiotherapy-treated glioma patients and non-radiotherapy treated glioma patients in the validated cohort of our study. G, SFRP2 expression in WHO grade II, III and IV glioma patients in the validated cohort of our study. H, overall survival of glioma patients according to SFRP2 expression in the validated cohort of our study. I, progression-free survival of glioma patients according to SFRP2 expression in the validated cohort of our study. J, IHC staining of SFRP2 in radiotherapy-treated glioma tissue and non-radiotherapy treated glioma tissue. K, SFRP2 expression in glioma cell lines or normal glia tissue was evaluated by qRT-PCR. **P< 0.001.

To search for potential genes involved in radioresistance of glioma patients, we analyzed the data downloaded from the TCGA database. A total of 263 glioma patients (grade II or grade III) from the TCGA database were enrolled. Among them 143 patients received radiotherapy, while the rest 120 patients did not. Limma package (version: 3.40.2) of R software was used to identify differentially expressed mRNAs between radiotherapy treated and non-radiotherapy treated glioma patients. The differentially expressed mRNAs were defined as adjusted *p*< 0.05 and |log_2_ Fold Change| ≥ 1, and depicted in the volcano plots (Fig 1A, S1 Table). A series of genes such as EN1, S100A4, ANXA1, PDPN, IGFBP2 and CHI3L1 were upregulated in radiotherapy treated glioma patients, while other genes such as PRLHR, SFRP2, BRINP1, TRIM67, LHX5, KCNIP2 and NSG2 were downregulated (S1 Table). In the present study, we focused on SFRP2 because it ranked the first in the downregulated genes (Fig 1A, S1 Table), and previous studies indicate that SFRP2 acts as a tumor-suppressor in glioma and regulates Wnt signaling activation in various cancers [17,20,21]. We found that SFRP2 was significantly downregulated in radiotherapy treated glioma patients compared with non-radiotherapy treated glioma patients (Fig 1B). The expression of SFRP2 was further analyzed in 662 glioma patients from the TCGA database. The expression of SFRP2 was negatively correlated with advanced tumor stage of glioma patients (Fig 1C). The SFRP2 level was dramatically decreased in glioblastoma patients (grade IV, n = 153) compared with patients with grade II (n = 248) or III (n = 261) gliomas (Fig 1C). Next, we divided the glioma patients (n = 662) into SFRP2 high expression or low expression group by using the median expression as cut-off value. Low SFRP2 expression was apparently correlated with poor overall-survival and progression-free survival of glioma patients (Fig 1D and 1E). The above results were analyzed from the TCGA database. We also validated these results in a cohort of glioma patients (n = 166) recruited from the Second Hospital of Shanxi Medical University in our study. These patients were radiotherapy treated (n = 88) or non-radiotherapy (n = 78) treated. We found that SFRP2 was evidently downregulated in radiotherapy treated glioma patients (Fig 1F), and low SFRP2 expression was associated with higher tumor stage (Fig 1G). In Kaplan-Meier analysis, patients with low SFRP2 expression had a poor overall-survival and progression-free survival (Fig 1H and 1I). The correlation between SFRP2 expression and clinicopathological characteristics of glioma patients was also evaluated. Our data indicated that low SFRP2 expression was positively correlated with larger tumor size, advanced tumor stage, radiotherapy treatment and radioresistance (Table 1). In IHC staining, non-radiotherapy treated glioma patients showed increasing number of SFRP2 positive cells compared with that in radiotherapy treated glioma patients (Fig 1J). The expression of SFRP2 in glioma cell lines was evaluated by qRT-PCR. We found that SFRP2 was high expressed in SW1783, U87MG, SW1088 and GMS-10 cell lines, and low expressed in LN18, T98G, GOS-3 and U251MG cell lines (Fig 1K). Taken together, the above results from the TCGA database and our validation cohort suggested that SFRP2 was downregulated in radiotherapy treated glioma patients, and low SFRP2 expression was correlated with higher tumor stage and poor prognosis.

**Table 1:** Correlation between SFRP2 expression and clinicopathological characteristics of glioma patients.

Characteristics	n	SFRP2 expression		*P* value
		Low	High	
Age (Years)				0.298
≤60	59	25	34	
>60	107	61	46	
Sex				0.685
Male	71	35	36	
Female	95	51	44	
Tumor size (mm)				**0.025**
≥ 30	105	71	34	
<30	61	15	46	
WHO grade				**0.001**
II	45	10	35	
III	60	29	31	
IV	61	47	14	
Treatment				**0.035**
Radiotherapy	78	53	25	
Non-Radiotherapy	88	33	55	
Radiotherapy response				**0.001**
Sensitive	29	12	17	
Resistant	49	41	8	

### SFRP2 knockdown promotes soft agar colony formation, cancer stemness and radioresistance of glioma cells

**Fig 2:**
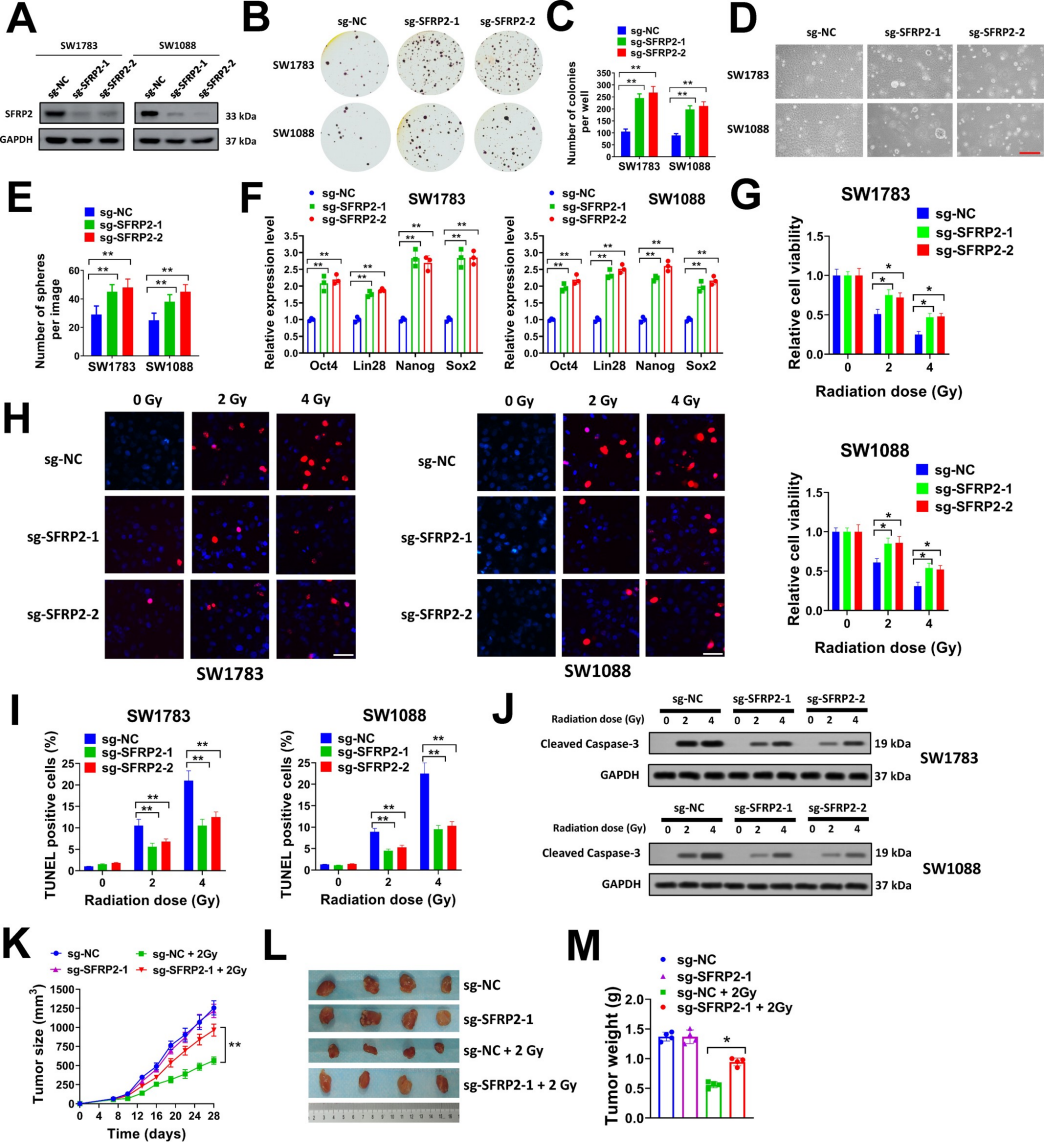
SFRP2 knockdown promotes soft agar colony formation, cancer stemness and radioresistance of glioma cells. A, SW1783 and SW1088 cells were introduced with sg-SFRP2-1, sg-SFRP2-2 or sg-NC lentiviral particles, then protein expression of SFRP2 was evaluated by western blot. B-C, SW1783 and SW1088 cells were introduced with sg-SFRP2-1, sg-SFRP2-2 or sg-NC lentiviral particles, then seeded in 6-well plates at 8000/well for soft agar assay. Representative plates (B) and average number of colonies per well (C) were shown. D-E, SW1783 and SW1088 cells were introduced with sg-SFRP2-1, sg-SFRP2-2 or sg-NC lentiviral particles, then seeded in 6-well plates at 3000/well for sphere formation assay. Representative images (D) and average number of spheres per image (E) were shown. F, expression of Oct4, Lin28, Nanog and Sox2 in SW1783 and SW1088 cells introduced with sg-SFRP2-1, sg-SFRP2-2 or sg-NC lentiviral particles was evaluated by qRT-PCR. G, SW1783 and SW1088 cells were introduced with sg-SFRP2-1, sg-SFRP2-2 or sg-NC lentiviral particles, then seeded in 96-well plates (3000/well) and treated with a single dose of 2 or 4 Gy X-ray irradiation. Cell viability was tested at 72 h after the irradiation. H-J, SW1783 and SW1088 cells infected with sg-SFRP2-1, sg-SFRP2-2 or sg-NC lentiviral particles were seeded in 6-well plates (1 × 10^6^/well) and treated with a single dose of 2 or 4 Gy X-ray irradiation. Then cells were used for TUNEL staining (H-I) or western blot (J) at 24 h after irradiation. Representative images (H) and percentages of TUNEL positive cells (I) were shown. K-M, SW1783 cells (2 × 10^6^) infected with sg-SFRP2-1 or sg-NC lentiviral particles were subcutaneously injected into nude mice. Tumor xenografts were treated with/without 2 Gy X-ray irradiation daily for three weeks from day 7. Tumor growth curves (K), image of tumor xenografts (L) and tumor weight (M) were shown. *P< 0.05, **P< 0.001.

The potential functions of SFRP2 in glioma cells were evaluated by loss-of-function assays. We used the CRISPR/Cas9 system to knock out SFRP2 in glioma cells. Two short guide RNAs (sgRNAs) specifically targeting SFRP2 were designed (sg-SFRP2-1 and sg-SFRP2-2). Our data indicated that sg-SFRP2-1 and sg-SFRP2-2 successfully depleted SFRP2 expression in SW1783 and SW1088 cells (Fig 2A). These two cell lines were selected in our study as they had high endogenous levels of SFRP2, thus we wanted to see the influence of SFRP2 knockdown on these cells. The tumor suppressive function of SFRP2 was evaluated by soft agar colony formation assay. SFRP2 knockdown by these two sgRNAs increased colony formation in SW1783 and SW1088 cells, indicating that SFRP2 knockdown promoted malignant transformation (Fig 2B and 2C). Cancer stemness plays an important role in intrinsic or acquired chemo- or radioresistance of glioma cells [7,12,13]. As SFRP2 was decreased by radiation treatment, we speculated that SFRP2 might affect cancer stemness of glioma cells. In sphere formation assay, SFRP2 knockdown evidently facilitated sphere formation of SW1783 and SW1088 cells when compared with cells introduced with sg-NC (Fig 2D and 2E). In addition, the expression of cancer stemness markers (Oct4, Lin28, Nanog, and Sox2) was obviously increased in SW1783 and SW1088 cells after SFRP2 knockdown (Fig 2F). CD133 is a marker for glioma stemness cells. In flow cytometry analysis, the percentage of CD133+ positive cells was dramatically increased after SFRP2 knockdown (S1A and S1B Fig). These results suggested that SFRP2 knockdown promoted cancer stemness of glioma cells. The influence of SFRP2 knockdown on radiosensitivity of glioma cells was evaluated. The cell viability of SW1783 and SW1088 cells was significantly suppressed by 2 or 4 Gy X-ray radiation, but this was evidently attenuated by SFRP2 knockdown (Fig 2G). Cell apoptosis of SW1783 and SW1088 cells after 2 or 4 Gy X-ray radiation was evaluated by TUNEL staining and flow cytometry. We found that the number of TUNEL positive cells were significantly increased after 2 or 4 Gy X-ray radiation in SW1783 and SW1088 cells, but this was weakened by SFRP2 knockdown (Fig 2H and 2I). In flow cytometry, the percentages of apoptotic cells were increased by 2 or 4 Gy X-ray radiation, but SFRP2 knockdown attenuated this effect (S1C and S1D Fig). Cleaved caspase-3 is a marker of cell apoptosis. In western blot analysis, SFRP2 knockdown reduced the expression level of cleaved caspase-3 in SW1783 and SW1088 cells that received with 2 or 4 Gy X-ray radiation (Fig 2J). These results indicated that SFRP2 knockdown increased radioresistance of glioma cells and reduced cell apoptosis after X-ray radiation *in vitro*. To evaluate the influence of SFRP2 on radiosensitivity *in vivo*, SW1783 cells (2 × 10^6^) transduced with sg-SFRP2-1 or sg-NC were subcutaneously injected into nude mice, then tumor xenografts were allowed to grow for 7 days. Next, tumor xenografts were gave radiation treatment (2 Gy) once daily for three weeks. In our study, radiation treatment significantly suppressed tumor xengraft growth of SW1783 cells transduced with sg-NC, but the inhibitory effect was weakened in SW1783 cells transduced with sg-SFRP2-1 (Fig 2K–2M). Collectively, our results indicated that SFRP2 knockdown promoted soft agar colony formation, cancer stemness and radioresistance of glioma cells *in vitro* and *in vivo*.

### Forced SFRP2 expression suppresses soft agar colony formation, cancer stemness and radioresistance of glioma cells

**Fig 3:**
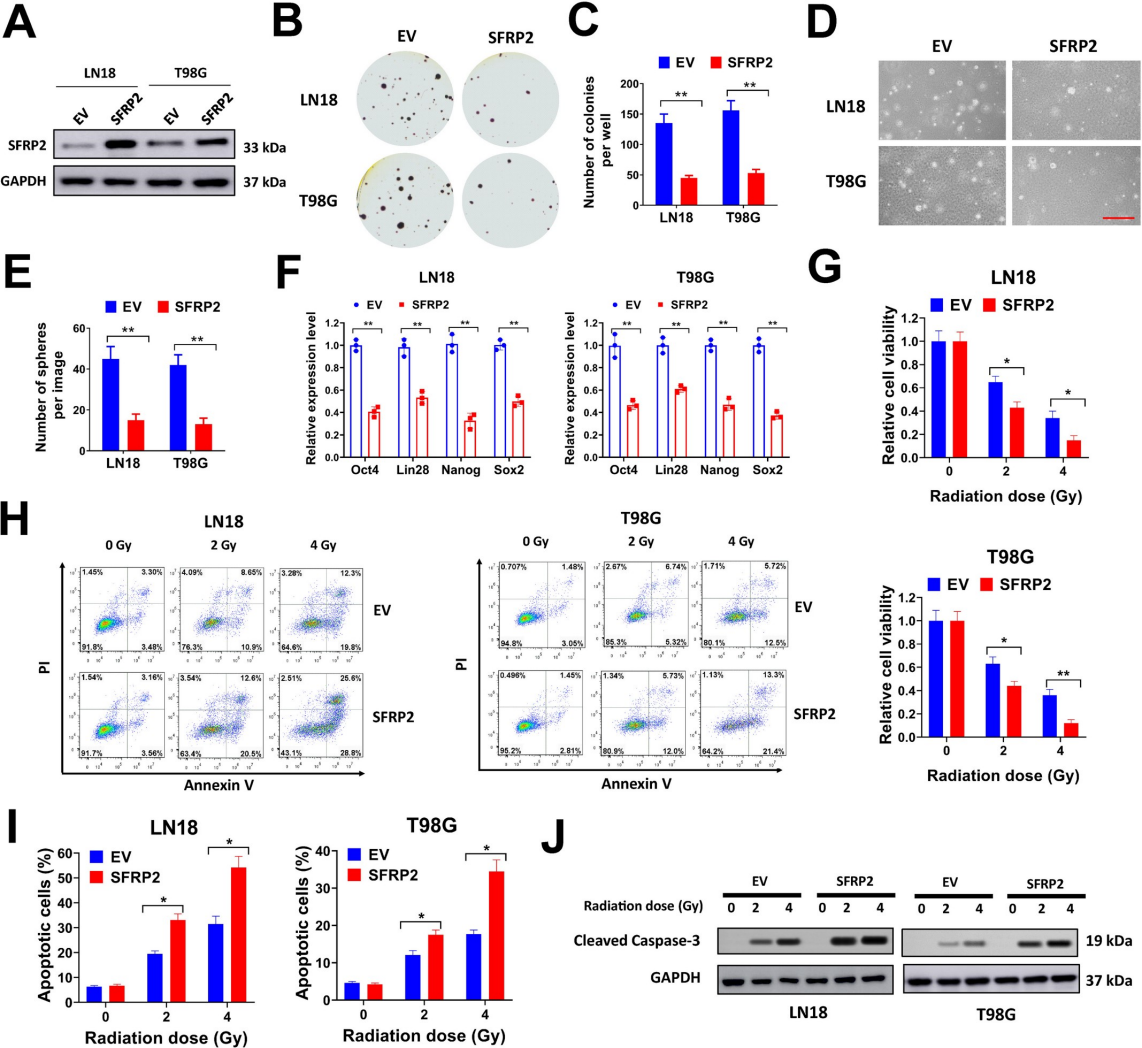
Forced SFRP2 expression suppresses soft agar colony formation, cancer stemness and radioresistance of glioma cells. A, LN18 and T98G cells were infected with SFRP2 expression lentivirus or empty vector (EV) control, then protein expression of SFRP2 was evaluated by western blot. B-C, LN18 and T98G cells were infected with SFRP2 expression lentivirus or EV control, then seeded in 6-well plates at 8000/well for soft agar assay. Representative plates (B) and average number of colonies per well (C) were shown. D-E, LN18 and T98G cells were infected with SFRP2 expression lentivirus or EV control, then seeded in 6-well plates at 3000/well for sphere formation assay. Representative images (D) and average number of spheres per image (E) were shown. F, expression of Oct4, Lin28, Nanog and Sox2 in LN18 and T98G cells infected with SFRP2 expression lentivirus or EV control was evaluated by qRT-PCR. G, LN18 and T98G cells were infected with SFRP2 expression lentivirus or EV control, then seeded in 96-well plates (3000/well) and treated with a single dose of 2 or 4 Gy X-ray irradiation. Cell viability was tested at 72 h after irradiation. H-J, LN18 and T98G cells infected with SFRP2 expression lentivirus or EV control were seeded in 6-well plates (1 × 10^6^/well) and treated with a single dose of 2 or 4 Gy X-ray irradiation. Then cells were used for flow cytometry (H-I) or western blot (J) at 24 h after the irradiation. Percentages of Annexin-V positive cells (I) were shown. *P< 0.05, **P< 0.001.

In the present study, we overexpressed SFRP2 in glioma cells and evaluated for the influence on colony formation, cancer stemness and radioresistance. LN18 and T98G cells were selected for overexpression studies due to their low endogenous levels of SFPR2. In our study, a SFRP2 expression lentivirus vector was constructed and introduced into LN18 and T98G cells. LN18 and T98G cells introduced with SFRP2 expression lentivirus showed evidently upregulation of SFRP2 protein expression (Fig 3A). Next, LN18 and T98G cells transduced with SFRP2 expression lentivirus were used for soft agar colony formation assay and sphere formation assay. Enforced SFRP2 expression significantly suppressed soft agar colony formation and sphere formation in LN18 and T98G cells compared with empty vector (EV) control (Fig 3B–3E). Additionally, SFRP2 overexpression reduced the percentage of CD133+ positive cells in LN18 and T98G (S2A and S2B Fig). Moreover, SFRP2 overexpression reduced the expression levels of cancer stemness markers such as Oct4, Lin28, Nanog, and Sox2 (Fig 3F). The influence of SFRP2 overexpression on glioma cells were evaluated. The cell viability of LN18 and T98G cells was reduced by 2 or 4 Gy X-ray radiation, and this effect was further enhanced by SFRP2 overexpression (Fig 3G). The apoptotic cells after radiation were evaluated by TUNEL staining and flow cytometry. In TUNEL staining, the number of TUNEL positive cells were increased after 2 or 4 Gy X-ray radiation in LN18 and T98G cells, but this was aggrandized by SFRP2 overexpression (S2C and S2D Fig). In flow cytometry, our data indicated that the percentages of Annexin V positive apoptotic cells were apparently increased in LN18 and T98G cells transduced with SFRP2 expression lentivirus compared with EV control (Fig 3H and 3I). The expression level of cleaved caspase-3 in LN18 and T98G cells was evaluated by western blot. We found that SFRP2 overexpression increased protein level of cleaved caspase-3 in LN18 and T98G cells (Fig 3J). Above all, our data indicated that forced SFRP2 expression suppressed soft agar colony formation, cancer stemness and radioresistance of glioma cells.

### SFRP2 regulates Wnt/β-catenin activation in glioma cells

**Fig 4:**
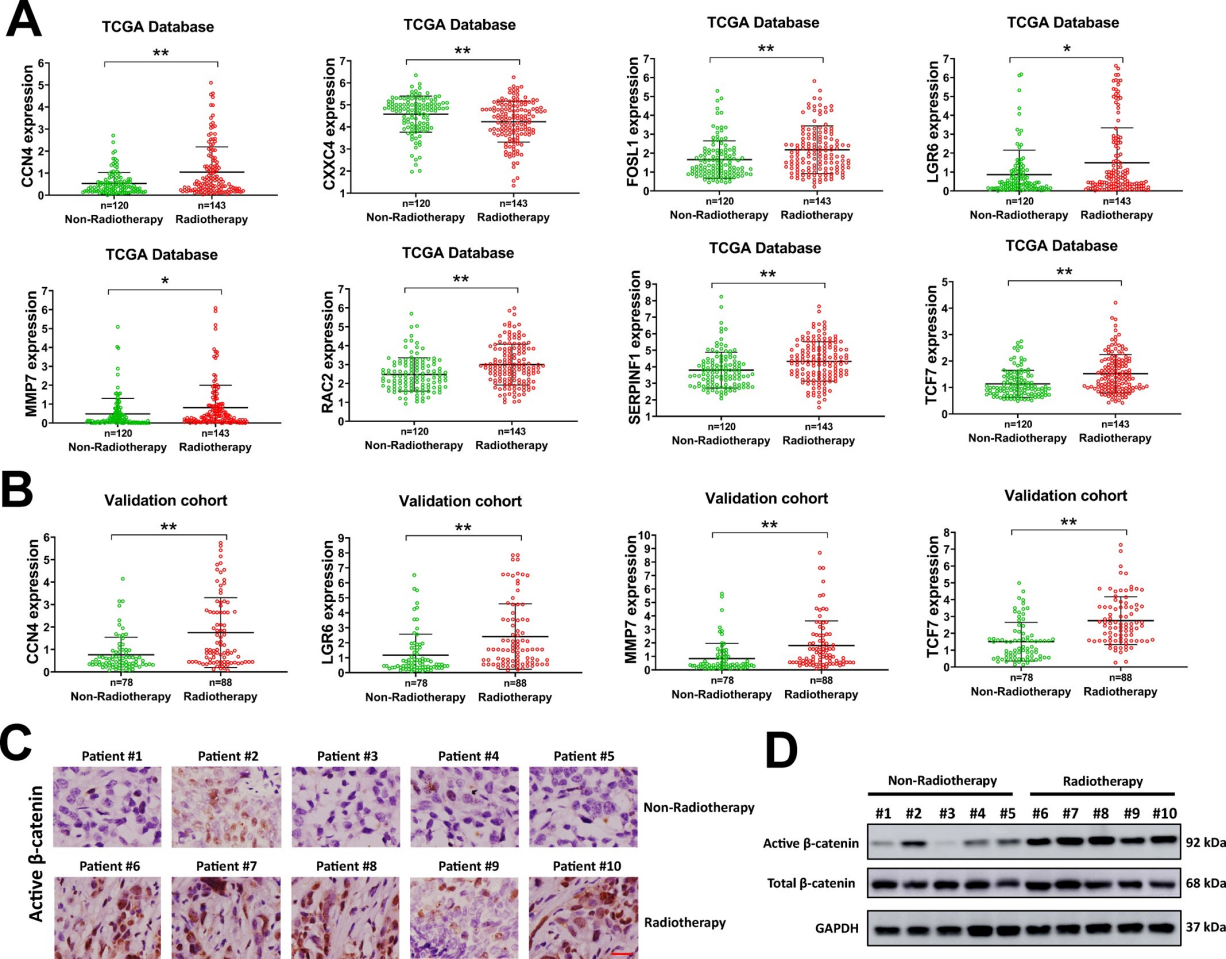
Wnt signaling is activated in radiotherapy treated glioma patients. A, the expression of indicated genes in TCGA database. B, the expression of indicated genes in the Validation cohort of our study. C-D, the expression of active β-catenin in radiotherapy treated and non-radiotherapy treated glioma samples was evaluated by IHC staining (C) or western blot (D). *P< 0.05, **P< 0.001.

**Fig 5:**
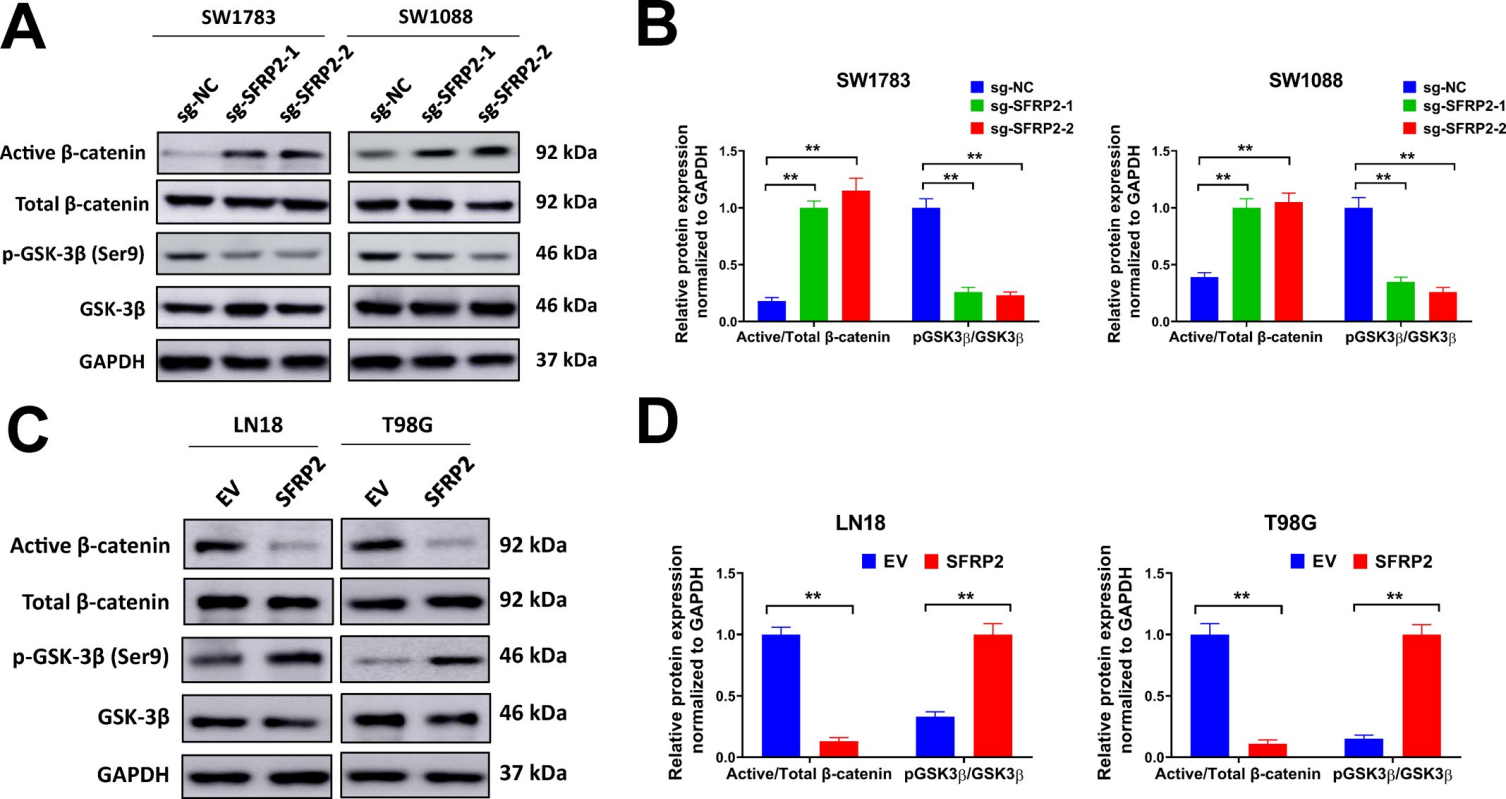
SFRP2 regulates Wnt/β-catenin activation in glioma cells. A-B, SW1783 and SW1088 cells were introduced with sg-SFRP2-1, sg-SFRP2-2 or sg-NC lentiviral particles, then protein expression of indicated genes was evaluated by western blot (A). Relative protein expression compared with GAPDH (B) was shown. C-D, LN18 and T98G cells were infected with SFRP2 expression lentivirus or EV control, then protein expression of indicated genes was evaluated by western blot (C). Relative protein expression compared with GAPDH (D) was shown. **P< 0.001.

SFRP2 is a key regulator of Wnt/β-catenin pathway, thus we evaluated the expression of Wnt pathway related genes in glioma patients. A number of Wnt pathway related genes were upregulated or downregulated in radiotherapy treated glioma patients from the TCGA database, such as LGR6, SERPINF1, RAC2, FOSL1, CCN4, PLCB2, JUN, WNT5A, TCF7, FZD2, FZD6, MMP7, ROR2, CXXC4 and SFRP1 (S1 Table). The expression levels of some of these genes were depicted in Fig 4A. Our data indicated that the Wnt pathway activation related genes such as CCN4, FOSL1, LGR6, MMP7, RAC2, SERPINF1 and TCF7 were upregulated, while Wnt pathway inactivation related gene such as CXXC4 was downregulated in radiotherapy treated glioma patients from TCGA database (Fig 4A). This was also validated in our study. In the validation cohort of glioma patients, we found that CCN4, LGR6, MMP7 and TCF7 were obviously upregulated (Fig 4B). Next, we evaluated the protein level of active β-catenin in glioma patients by IHC staining and western blot. In IHC staining, the number of active β-catenin positive cells were significantly increased in radiotherapy treated glioma patients compared with non-radiotherapy treated patients (Fig 4C). In western blot analysis, the protein level of active β-catenin was apparently upregulated in radiotherapy treated glioma patients, too (Fig 4D). These data indicated that Wnt signaling was activated in radiotherapy treated glioma patients. The influence of SFRP2 on Wnt signaling activation was explored. We found that SFRP2 knockdown increased the protein level of active β-catenin and reduced the phosphorylation of GSK-3β in SW1783 and SW1088 cells (Fig 5A and 5B). Meanwhile, the protein level of active β-catenin was decreased while phosphorylated GSK-3β elevated by SFRP2 overexpression in LN18 and T98G cells (Fig 5C and 5D). As SFRP2 was dramatically decreased in radiotherapy treated glioma patients, we speculated that downregulation of SFRP2 might facilitate Wnt signaling activation in these glioma patients. Taken together, our data indicated that Wnt signaling was activated in radiotherapy treated glioma patients, and this might due to downregulation of SFRP2.

### Pharmacological inhibition of Wnt/β-catenin signaling abrogates the effects of SFRP2 knockdown on soft agar colony formation, cancer stemness and radioresistance of glioma cells

**Fig 6:**
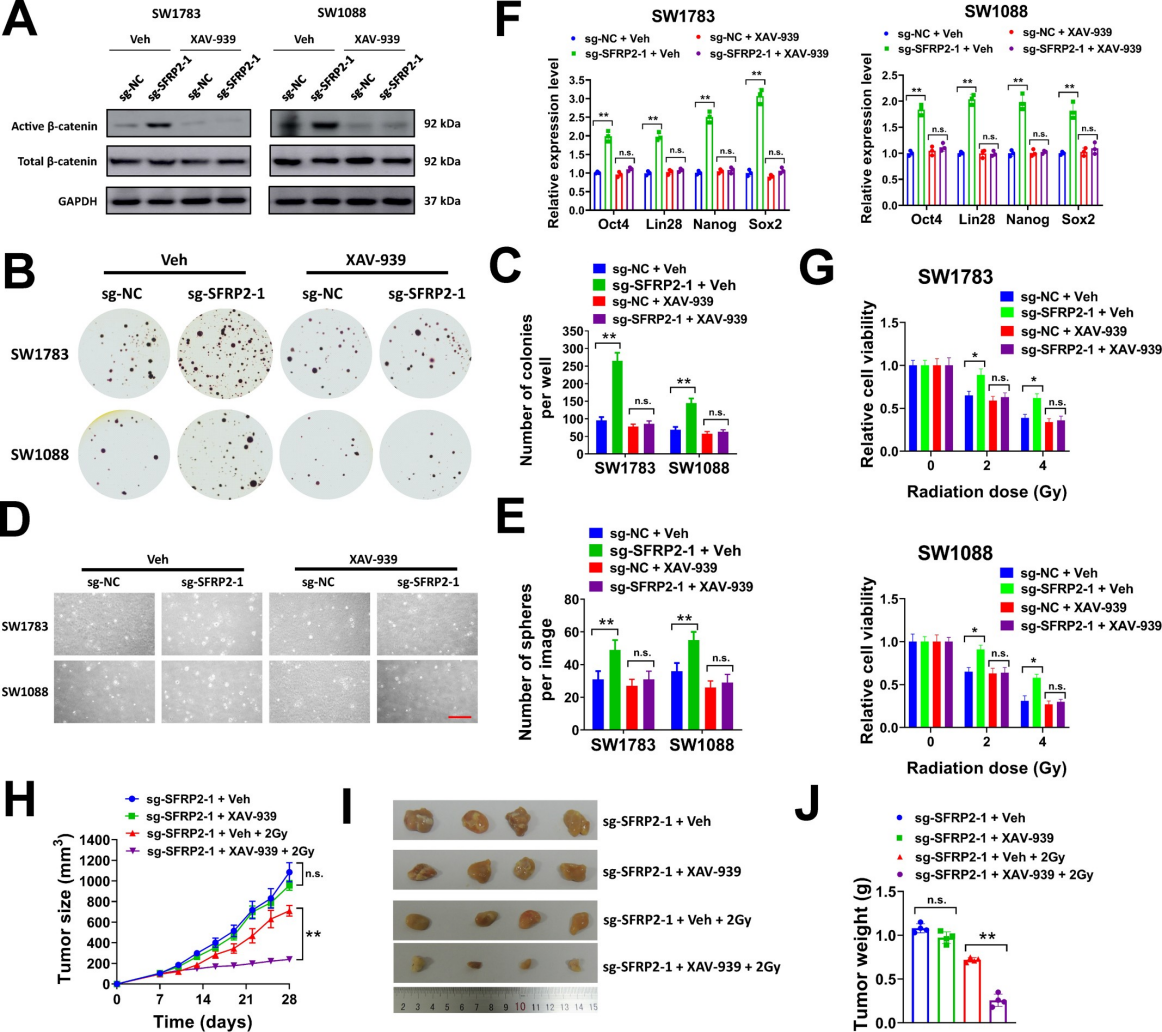
Pharmacological inhibition of Wnt/β-catenin pathway represses sphere formation, stemness and radioresistance of glioma cells. A, SW1783 and SW1088 cells infected with sg-SFRP2-1 or sg-NC lentiviral particles were seeded in 6-well plates (1 × 10^6^/well), and treated with 1 μM XAV-939 or equal volume of DMSO (Veh) for 24 h. The protein expression of indicated genes was evaluated by western blot. B-C, SW1783 and SW1088 cells were introduced with sg-SFRP2-1 or sg-NC lentiviral particles, then seeded in 6-well plates at 8000/well for soft agar assay. Cells were treated with 1 μM XAV-939 or equal volume of DMSO (Veh) at the same time. Representative plates (B) and average number of colonies per well (C) were shown. D-E, SW1783 and SW1088 cells were introduced with sg-SFRP2-1 or sg-NC lentiviral particles, then seeded in 6-well plates at 3000/well for sphere formation assay. Cells were treated with 1 μM XAV-939 or equal volume of DMSO (Veh) at the same time. Representative images (D) and average number of spheres per image (E) were shown. F, SW1783 and SW1088 cells introduced with sg-SFRP2-1 or sg-NC lentiviral particles were treated with 1 μM XAV-939 or equal volume of DMSO (Veh) for 6 d, then the expression of Oct4, Lin28, Nanog and Sox2 was evaluated by qRT-PCR. G, SW1783 and SW1088 cells introduced with sg-SFRP2-1 or sg-NC lentiviral particles were seeded in 96-well plates (3000/well) and treated with 2 or 4 Gy X-ray irradiation, 1 μM XAV-939 or equal volume of DMSO (Veh) as indicated. Cell viability was tested at 72 h after the irradiation. H-J, SW1783 cells (2 × 10^6^) infected with sg-SFRP2-1 or sg-NC lentiviral particles were subcutaneously injected into nude mice. Tumor xenografts were treated with 2 Gy X-ray irradiation, 25 mg/kg XAV-939 or equal volume of 50% PEG-400 as indicated daily for three weeks from day 7. Tumor growth curves (H), image of tumor xenografts (I) and tumor weight (J) were shown. *P< 0.05, **P< 0.001. n.s., not significant.

To determine whether the effect of SFRP2 in glioma cells was mediated by Wnt/β-catenin signaling, we used a Wnt/β-catenin pathway inhibitor XAV-939 to restrain Wnt/β-catenin signaling activation in glioma cells. As shown in Fig 6A, knockdown of SFRP2 increased the protein level of active β-catenin in SW1783 and SW1088 cells, but XAV-939 treatment completely inhibited β-catenin activation. Next, we evaluated the effect of Wnt/β-catenin inhibition in soft agar assay and sphere formation assay. Our data indicated that SW1783 and SW1088 cells introduced with sg-SFRP2-1 lentivirus formed more colonies than cells introduced with sg-NC, but exposing to XAV-939 entirely abolished this effect (Fig 6B and 6C). In sphere formation assay, XAV-939 treatment abrogated the effect of SFRP2 knockdown on sphere formation of SW1783 and SW1088 cells, too (Fig 6D and 6E). The expression of cancer stemness marker was evaluated. Our data suggested that SFRP2 knockdown promoted the expression of stemness markers such as Oct4, Lin28, Nanog, and Sox2, but XAV-939 treatment totally eliminated this effect (Fig 6F). Next, we evaluated the influence of XAV-939 on radioresistance of SW1783 and SW1088 cells. The cell viability of SW1783 and SW1088 cells was significantly suppressed by 2 or 4 Gy X-ray radiation, but this was partially abrogated by SFRP2 knockdown (Fig 6G). However, XAV-939 treatment revoked the effect of SFRP2 knockdown in X-ray radiated SW1783 and SW1088 cells. In tumor xenograft model, SW1783 cells (2 × 10^6^) transduced with sg-SFRP2-1 were subcutaneously injected into nude mice, then tumor xenografts were allowed to grow for 7 days. Next, mice were treated with XAV-939 and/or 2 Gy X-ray radiation once daily for three weeks. We found that XAV-939 treatment showed no apparently influence on tumor growth of SW1783 cells transduced with sg-SFRP2-1, while 2 Gy X-ray radiation exhibited moderate inhibitory effect on tumor growth (Fig 6H–6J). However, the tumor growth of SW1783 cells was significantly inhibited by the combination of XAV-939 treatment and 2 Gy X-ray radiation (Fig 6H–6J). Collectively, our data indicated that pharmacological inhibition of Wnt/β-catenin signaling abrogated the effects of SFRP2 knockdown on soft agar colony formation, cancer stemness and radioresistance of glioma cells.

## Discussion

The available evidences suggest that SFRP2 can be oncogenic or tumor suppressive in cancers, which is quite contradictory. The oncogenic role of SFRP2 is proved in osteosarcoma and renal cancer. For example, overexpression of SFRP2 promotes migration, invasion and metastasis of osteosarcoma cells *in vitro* and *in vivo* [23]. In renal cancer, enforced SFRP2 expression facilitates proliferation and *in vivo* tumor growth of renal cancer cells by activating canonical Wnt signaling [24]. In addition, SFRP2 is also upregulated and associated with poor prognosis of breast cancer and colorectal cancer [25,26]. On the contrary, SFRP2 is demonstrated to act as tumor suppressor in many other cancers, too. In gastric cancer, SFRP2 is downregulated in gastric cancer tissues, while forced SFRP2 expression suppresses proliferation and tumor growth of gastric cancer cells [27]. Loss of SFRP2 expression is also found in oral squamous cell carcinoma, and SFRP2 overexpression inhibits proliferation, cell cycle progression, and tumor growth by increasing the expression of GSK-3β and β-catenin [28]. Moreover, SFRP2 is proved to downregulate in a variety of cancers by promoter hypermethylation, including glioma [17]. In the present study, we found that SFRP2 was downregulated in radiotherapy treated glioma patients and correlated with advanced tumor stage and poor survival. Furthermore, SFRP2 knockdown promoted soft agar colony formation, cancer stemenss and radioresistance of glioma cells, while overexpression of SFRP2 showed contrary effects. Our data suggested that SFRP2 acted as a tumor suppressor in glioma. Though a previous report suggest that SFPR2 promotes clonogenicity and tumor growth of glioma cells [29], accumulated evidences reveal a tumor suppressive role of SFRP2 in glioma [21,30]. SFRP2 is frequently downregulated by promoter hypermethylation in glioma patients [20,31]. Mingzhi Han et al. demonstrate that SFPR2 is downregulated in glioma patients and predicts poor overall survival and progression-free survival, while overexpression of SFRP2 suppresses sphere formation, invasion and tumor growth of glioblastoma cells by regulating Wnt/β-catenin signaling [21]. In a recent study, SFRP2 is found to inhibit sphere formation, cancer stemness and proliferation of glioblastoma cells by acting as an antagonist of SOX2 [30]. These studies and our results all demonstrated that SFRP2 exerted tumor suppressive function in glioma.

In our study, we found that SFRP2 was the most significantly downregulated gene in radiotherapy treated glioma patients. In addition, SFRP2 knockdown promoted radioresistance of glioma cells and reduced cell apoptosis induced by X-ray irradiation, while overexpression of SFRP2 exhibited opposite effects. Our results for the first time demonstrated that SFRP2 was involved in radioresistance of glioma patients. Radiotherapy is frequently used for glioma treatment, however the effect is limited due to intrinsic or acquired resistance [32]. There are increasing evidences proving that the existence of cancer stem cells can facilitate radioresistance of glioma cells [33]. For instance, the CD133+ positive glioma stem cells are enriched after radiation, and exhibit resistance to radiotherapy by repairing radiation-induced DNA damage [13]. Moreover, Wenjuan Wang et al. prove that Cathepsin L and CD133 double positive glioma stem cells are extraordinarily radioresistance [34]. In our study, we found that SFRP2 knockdown promoted sphere formation and expression of cancer stemness markers, while overexpression of SFRP2 reduced sphere formation and expression of cancer stemness markers. These results suggested that downregulation of SFRP2 promoted cancer stemness, and this might explain the effect of SFRP2 in radioresistance of glioma cells. In addition, we found that SFRP2 regulated Wnt/β-catenin activation, and pharmacological inhibition of Wnt/β-catenin signaling by XAV-939 abrogated the effects of SFRP2 knockdown on cancer stemness and radioresistance in glioma. These results indicated that the effect of SFRP2 on cancer stemness and radioresistance was mainly due to regulating Wnt/β-catenin signaling. Indeed, numerous studies suggest that Wnt/β-catenin signaling is involved in cancer stemness and radioresistance of glioma [35]. For example, enhanced canonical Wnt signaling by R-spodin2 is proved to facilitate self-renewal and maintaining stem cell traits of glioblastoma cells [36]. Besides, Yonghyun Kim et al. find that Wnt signaling is activated in U373MG and GBM578 cells after *in vivo* ionizing radiation treatment [37]. These cells exhibit enhanced clonogenic and stem cell-like properties.

In the present study, we found that SFRP2 acted as an antagonist for canonical Wnt/β-catenin signaling in glioma cells. The members of SFRP family are firstly found to be antagonist of Wnt signaling for their ability to sequestrate Wnt ligands. For instance, SFRP2 can suppress Wnt3a-mediated canonical Wnt signaling by sequestering Wnt ligands and hindering their binding with frizzled receptors [38]. However, later studies propose an agonistic effect of the SFRP2 on Wnt signaling by directing binding to Frizzled receptors or affecting Wnt activation of soluble Wnt ligands [17]. The antagonistic or agonistic function of SFRP2 may depend on its expression level. Seham Skah et al. find that low concentration of SFRP2 activates Wnt signaling in murine intestinal epithelium COS7 cells while high concentration of SFRP2 shows inhibitory effect [39]. As SFRP2 was obviously downregulated in radiotherapy treated glioma patients, we speculated that the changing of SFPR2 expression level might correlated with Wnt signaling activation in these patients. Our data also indicated that SFRP2 acted as an antagonist but not agonist for Wnt/β-catenin signaling in glioma cells, and these results are supported by other studies, too. For example, Mingzhi Han et al. demonstrate that SFRP2 suppresses Wnt/β-catenin activity in glioblastoma cells [21]. Min Guo et al. find that SFRP2 induces a mesenchymal transition of glioblastoma cells by inhibiting non-canonical Wnt/β-catenin activity [30].

In summary, we found that SFRP2 was downregulated in radiotherapy treated glioma patients, and low SFRP2 expression was associated with advanced tumor stage, larger tumor size, radioresistance and poor survival. Knockdown of SFRP2 promoted soft agar colony formation, cancer stemness and radioresistance of glioma cells, while overexpression of SFRP2 showed opposite effects. In addition, Wnt/β-catenin signaling was activated in radiotherapy treated glioma patients. SFRP2 knockdown activated Wnt/β-catenin signaling in glioma cells, while overexpression of SFRP2 inhibited Wnt/β-catenin activation. Furthermore, we found that pharmacological inhibition of Wnt/β-catenin signaling abrogated the effects of SFRP2 knockdown on soft agar colony formation, cancer stemness and radioresistance of glioma cells. Our results for the first time demonstrated a role of SFRP2 in radioresistance of glioma cells, and suggested that inhibition of Wnt/β-catenin signaling might be a potential strategy for increasing radiosensitivity of glioma patients.

## Supporting information

S1 Fig SFRP2 knockdown promotes cancer stemness and radioresistance of glioma cells. A-B, SW1783 and SW1088 cells were introduced with sg-SFRP2-1, sg-SFRP2-2 or sg-NC lentiviral particles, then CD133+ positive cells were evaluated by flow cytometry (A). The percentage of CD133+ positive cells were shown (B). C-D, SW1783 and SW1088 cells infected with sg-SFRP2-1, sg-SFRP2-2 or sg-NC lentiviral particles were seeded in 6-well plates (1 × 10^6^/well) and treated with a single dose of 2 or 4 Gy X-ray irradiation. Then cells were stained with Annexin-V FITC and PI for flow cytometry (C). The percentages of Annexin-V FITC positive cells were shown (D). *P< 0.05.
(JPG)

S2 Fig Forced SFRP2 expression suppresses cancer stemness and radioresistance of glioma cells. A-B, LN18 and T98G cells were infected with SFRP2 expression lentivirus or empty vector (EV) control, then CD133+ positive cells were evaluated by flow cytometry (A). The percentage of CD133+ positive cells were shown (B). C-D, LN18 and T98G cells infected with SFRP2 expression lentivirus or empty vector (EV) control were seeded in 6-well plates (1 × 10^6^/well) and treated with a single dose of 2 or 4 Gy X-ray irradiation. Then cells were used for TUNEL staining at 24 h after irradiation. Representative images (C) and percentages of TUNEL positive cells (D) were shown. *P< 0.05, **P< 0.001.
(JPG)

S1 Table The differentially expressed mRNAs between radiotherapy treated and non-radiotherapy treated glioma patients in TCGA. (XLSX)

S1 File (PDF)
