# Supplementary figures and images for: Downregulation of SFRP2 facilitates cancer stemness and radioresistance of glioma cells via activating Wnt/β-catenin signaling
Source: PLoS One. 2021 Dec 1;16(12):e0260864. doi: 10.1371/journal.pone.0260864 (PMC8635357; doi:10.1371/journal.pone.0260864)

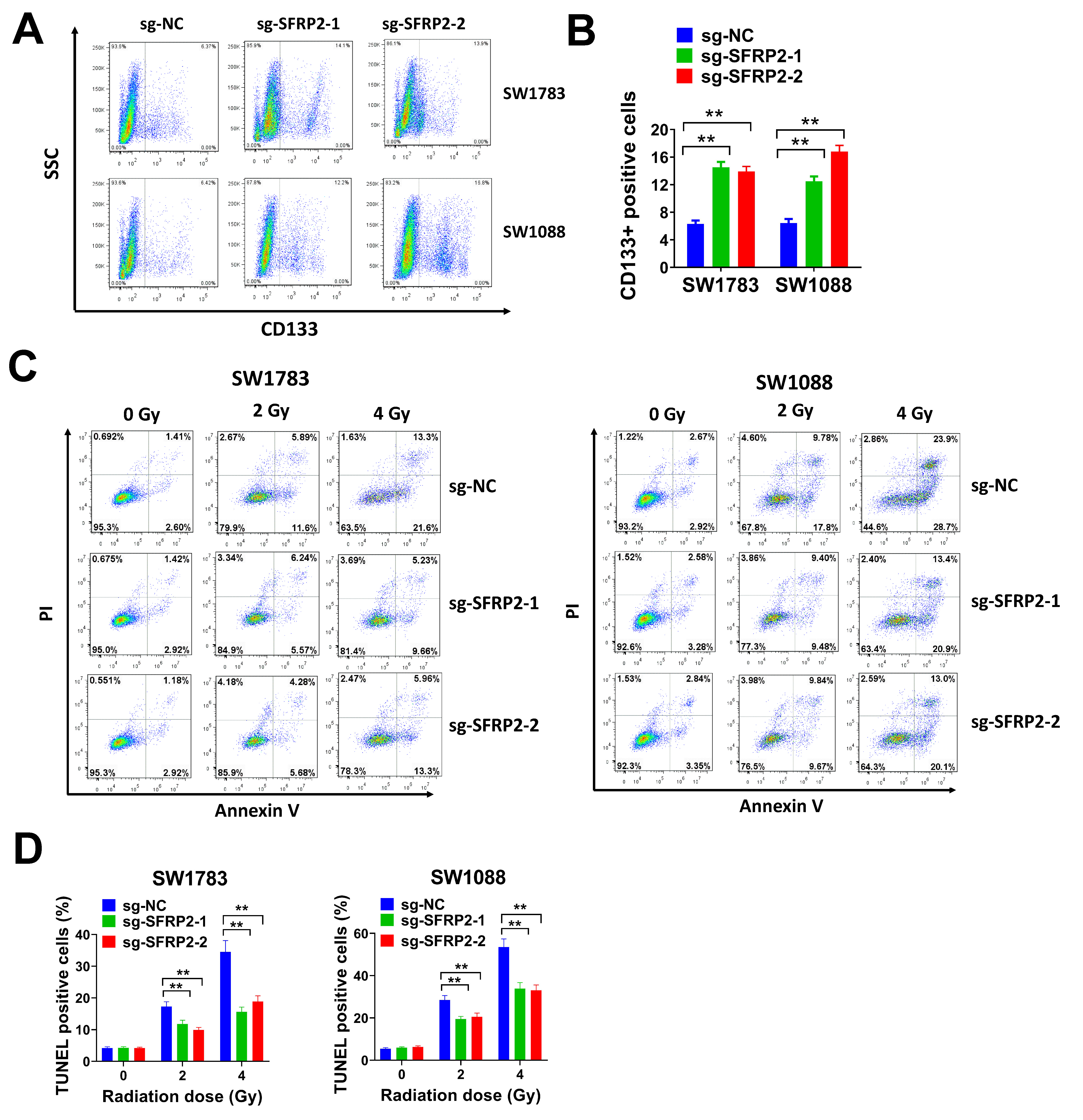

Supplement: S1 Fig — A-B, SW1783 and SW1088 cells were introduced with sg-SFRP2-1, sg-SFRP2-2 or sg-NC lentiviral particles, then CD133+ positive cells were evaluated by flow cytometry (A). The percentage of CD133+ positive cells were shown (B). C-D, SW1783 and SW1088 cells infected with sg-SFRP2-1, sg-SFRP2-2 or sg-NC lentiviral particles were seeded in 6-well plates (1 × 10^6^/well) and treated with a single dose of 2 or 4 Gy X-ray irradiation. Then cells were stained with Annexin-V FITC and PI for flow cytometry (C). The percentages of Annexin-V FITC positive cells were shown (D). *P< 0.05. (JPG) [file pone.0260864.s001.jpg]

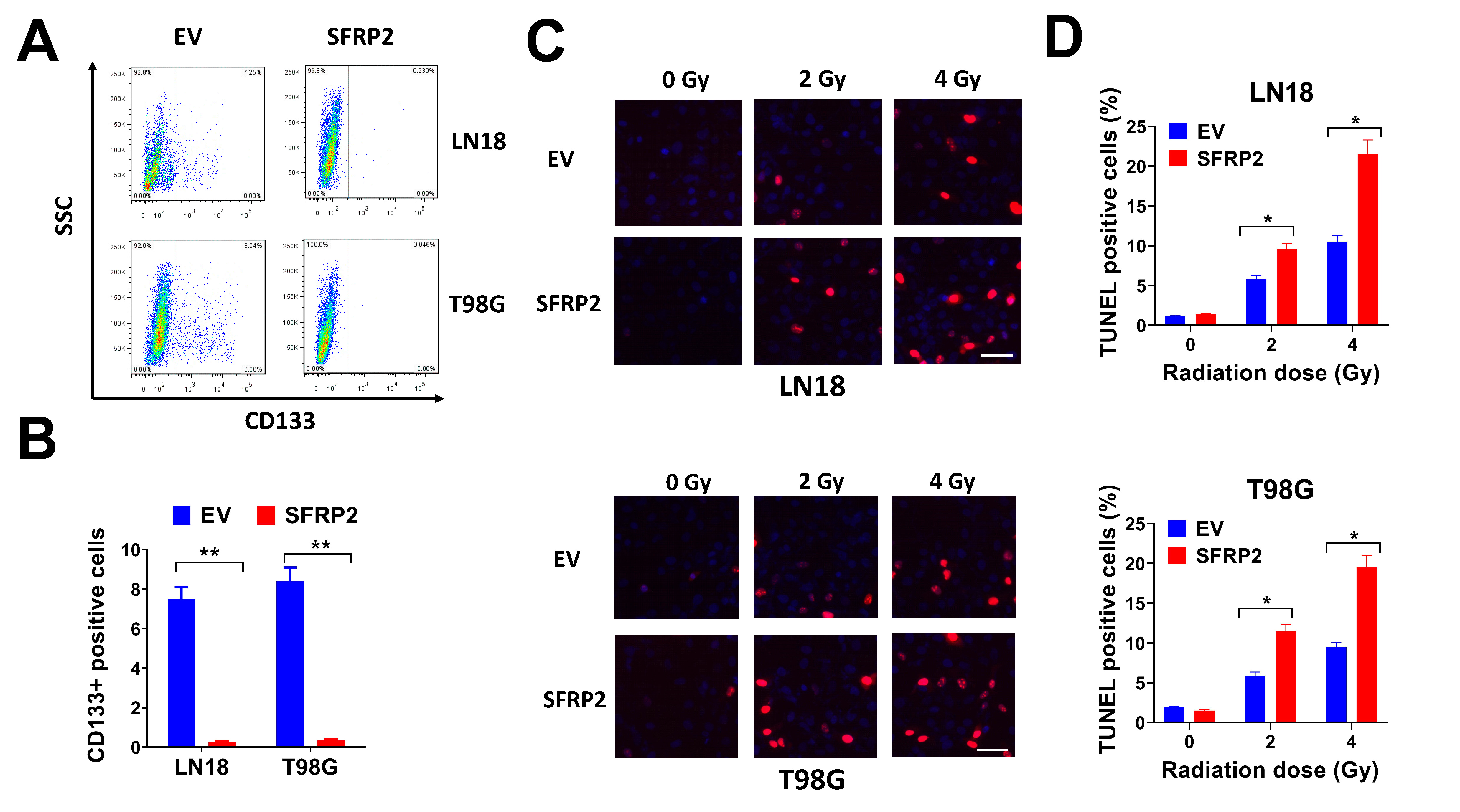

Supplement: S2 Fig — A-B, LN18 and T98G cells were infected with SFRP2 expression lentivirus or empty vector (EV) control, then CD133+ positive cells were evaluated by flow cytometry (A). The percentage of CD133+ positive cells were shown (B). C-D, LN18 and T98G cells infected with SFRP2 expression lentivirus or empty vector (EV) control were seeded in 6-well plates (1 × 10^6^/well) and treated with a single dose of 2 or 4 Gy X-ray irradiation. Then cells were used for TUNEL staining at 24 h after irradiation. Representative images (C) and percentages of TUNEL positive cells (D) were shown. *P< 0.05, **P< 0.001. (JPG) [file pone.0260864.s002.jpg]

**Figure 2A**

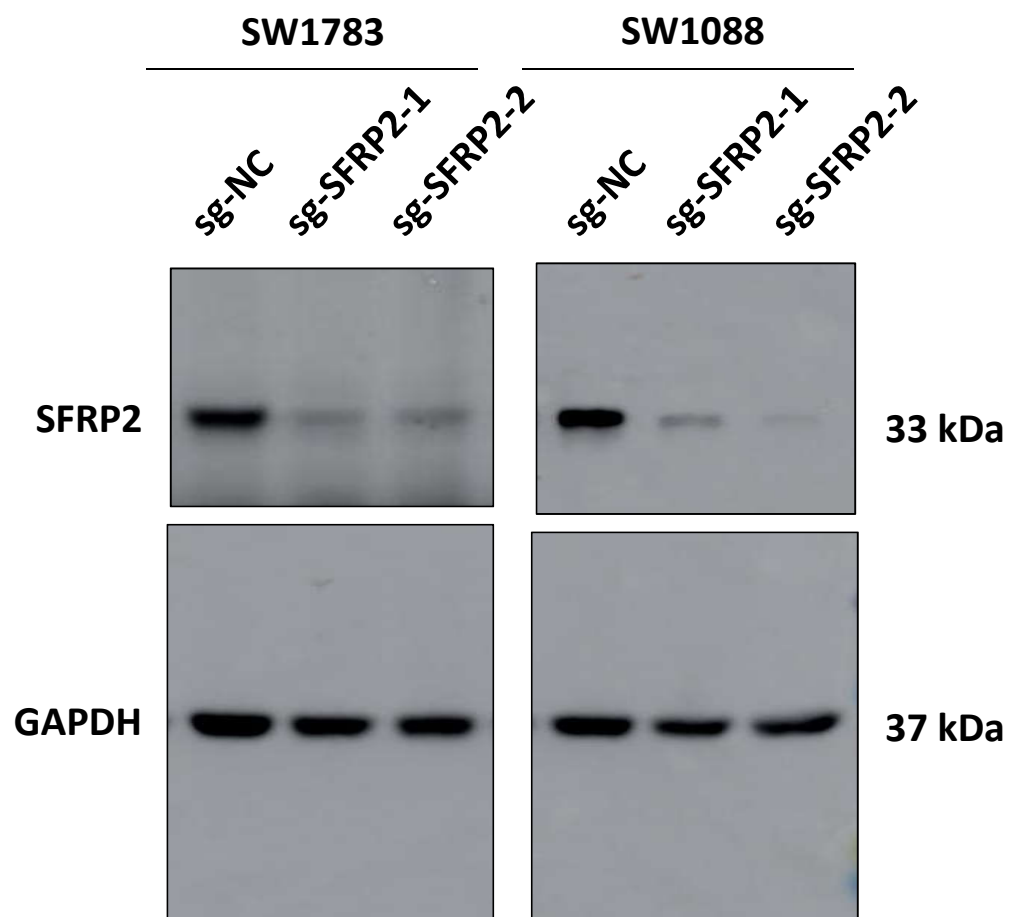

Figure 2J

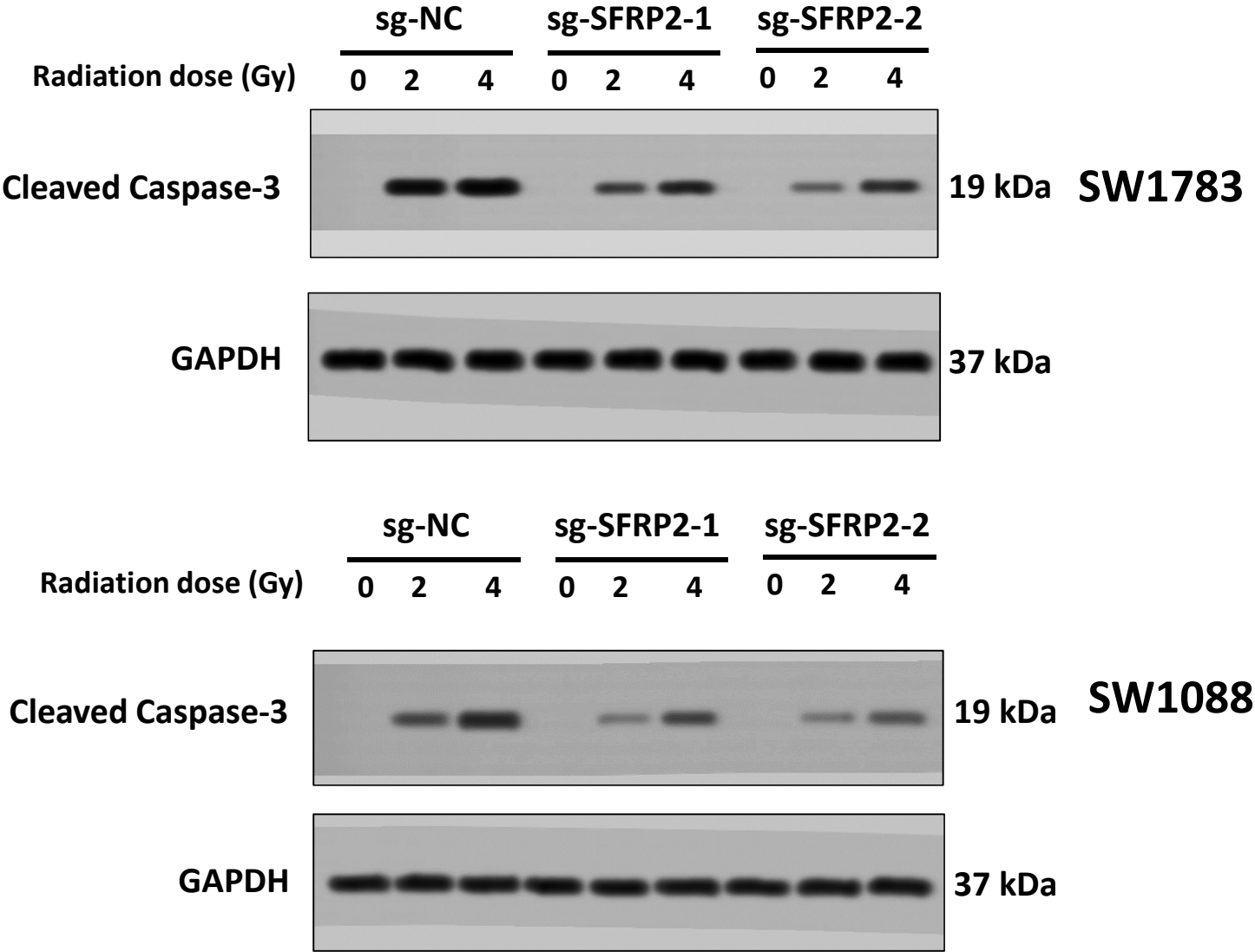

Figure 3A

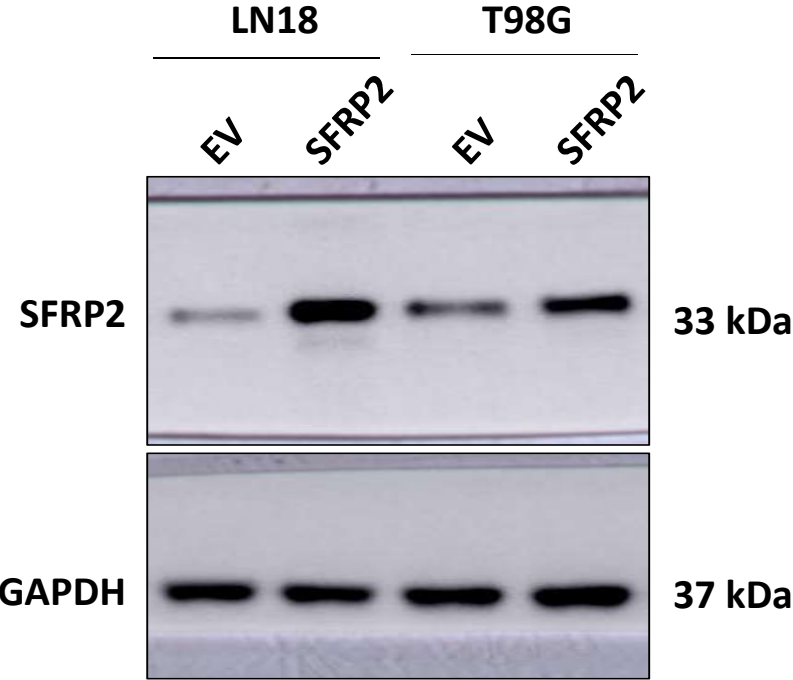

Figure 3J

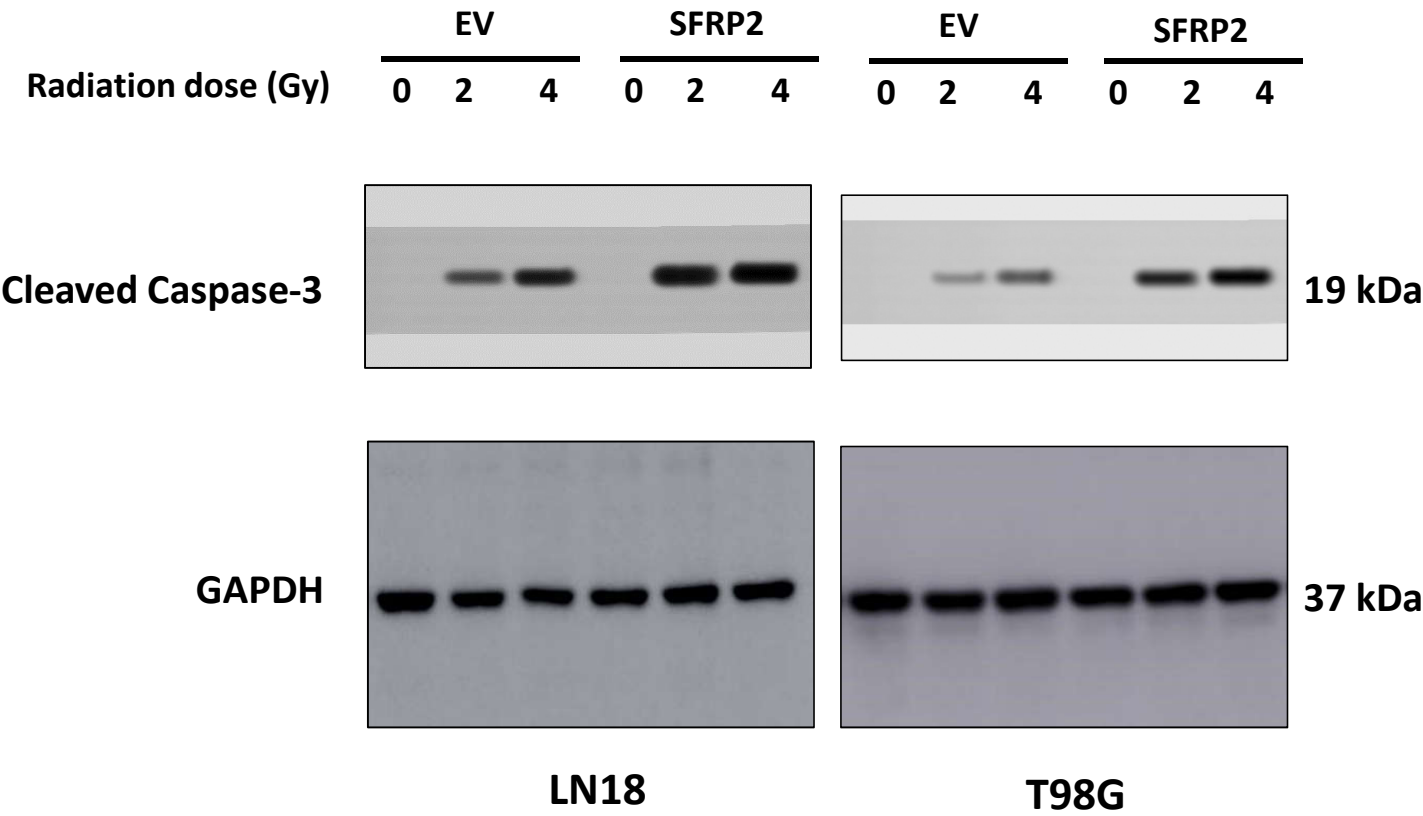

Figure 1E

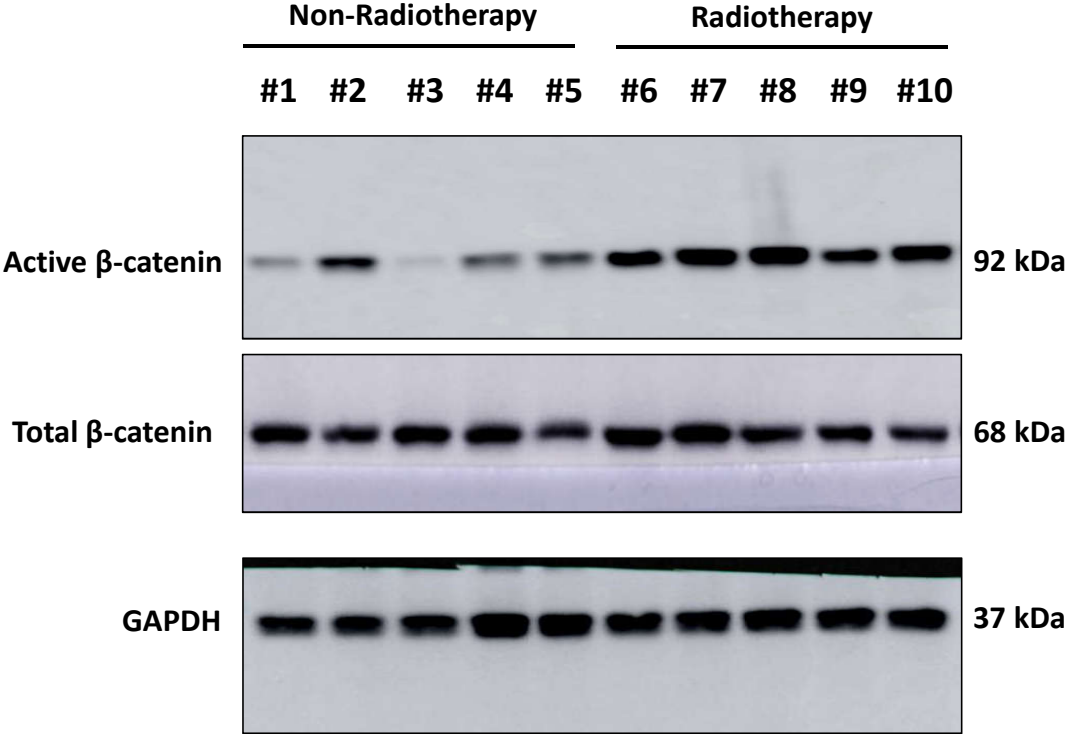

Figure 5A

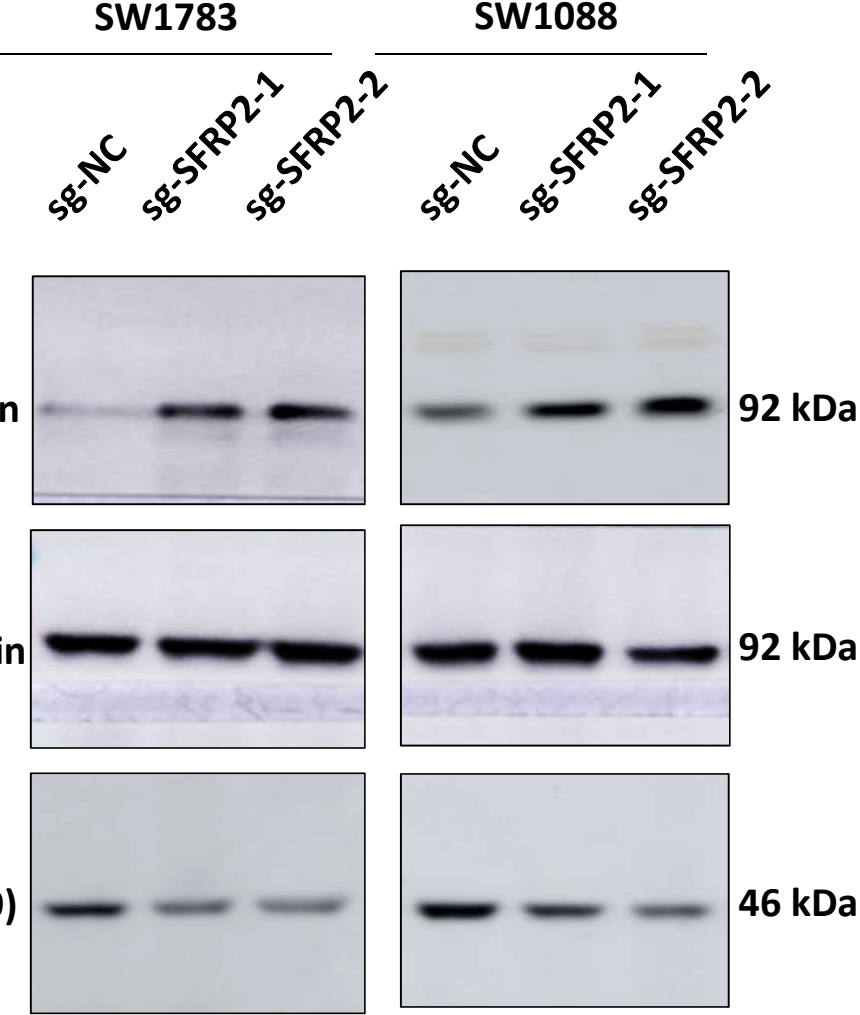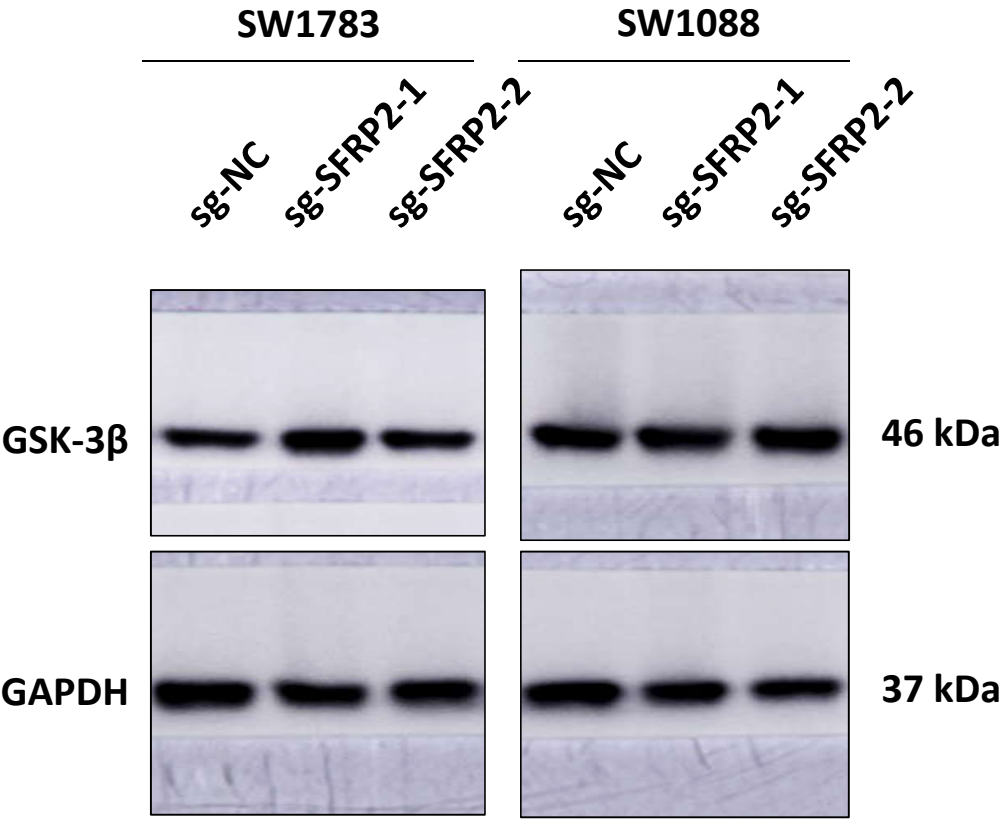

Figure 5C

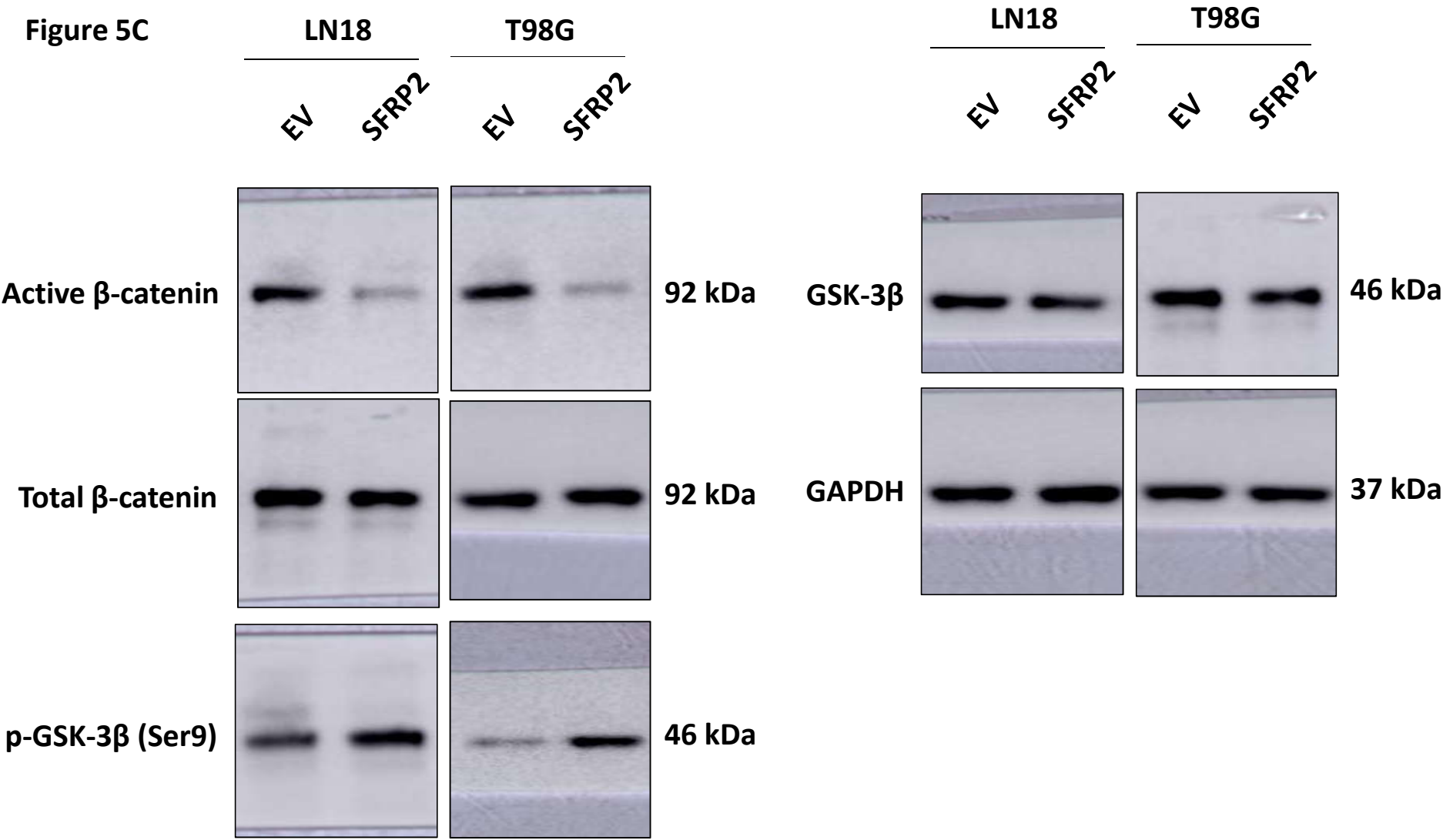

Figure 6A

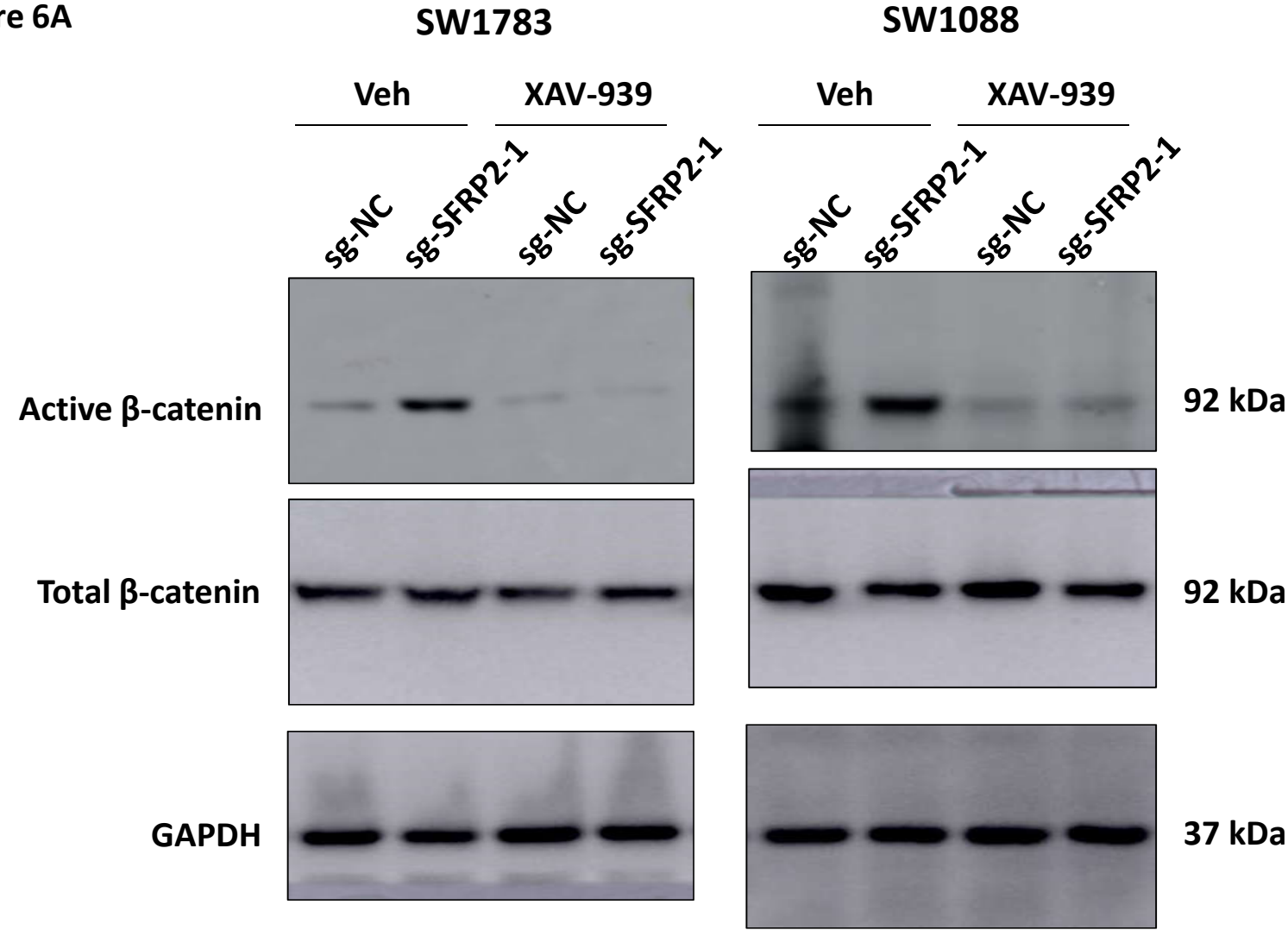

Supplement: S1 File — (PDF) [file pone.0260864.s004.pdf]
